# Difference analysis and characteristics of incompatibility group plasmid replicons in gram-negative bacteria with different antimicrobial phenotypes in Henan, China

**DOI:** 10.1186/s12866-024-03212-9

**Published:** 2024-02-19

**Authors:** Ruyan Chen, Chenyu Li, Haoyu Ge, Jie Qiao, Lei Fang, Cailin Liu, Jianjun Gou, Xiaobing Guo

**Affiliations:** Department of Laboratory Medicine, The First Affiliated Hospital of Zhengzhou University, Zhengzhou, China

**Keywords:** Plasmid typing, Incompatibility groups, Gram-negative bacteria, Carbapenem resistance

## Abstract

**Background:**

Multi-drug-resistant organisms (MDROs) in gram-negative bacteria have caused a global epidemic, especially the bacterial resistance to carbapenem agents. Plasmid is the common vehicle for carrying antimicrobial resistance genes (ARGs), and the transmission of plasmids is also one of the important reasons for the emergence of MDROs. Different incompatibility group plasmid replicons are highly correlated with the acquisition, dissemination, and evolution of resistance genes. Based on this, the study aims to identify relevant characteristics of various plasmids and provide a theoretical foundation for clinical anti-infection treatment.

**Methods:**

330 gram-negative strains with different antimicrobial phenotypes from a tertiary hospital in Henan Province were included in this study to clarify the difference in incompatibility group plasmid replicons. Additionally, we combined the information from the PLSDB database to elaborate on the potential association between different plasmid replicons and ARGs. The VITEK mass spectrometer was used for species identification, and the VITEK-compact 2 automatic microbial system was used for the antimicrobial susceptibility test (AST). PCR-based replicon typing (PBRT) detected the plasmid profiles, and thirty-three different plasmid replicons were determined. All the carbapenem-resistant organisms (CROs) were tested for the carbapenemase genes.

**Results:**

21 plasmid replicon types were detected in this experiment, with the highest prevalence of IncFII, IncFIB, IncR, and IncFIA. Notably, the detection rate of IncX3 plasmids in CROs is higher, which is different in strains with other antimicrobial phenotypes. The number of plasmid replicons they carried increased with the strain resistance increase. *Enterobacterales* took a higher number of plasmid replicons than other gram-negative bacteria. The same strain tends to have more than one plasmid replicon type. IncF-type plasmids tend to be associated with MDROs. Combined with PLSDB database analysis, IncFII and IncX3 are critical platforms for taking *bla*_KPC−2_ and *bla*_NDM_.

**Conclusions:**

MDROs tend to carry more complex plasmid replicons compared with non-MDROs. The plasmid replicons that are predominantly prevalent and associated with ARGs differ in various species. The wide distribution of IncF-type plasmids and their close association with MDROs should deserve our attention. Further investigation into the critical role of plasmids in the carriage, evolution, and transmission of ARGs is needed.

**Supplementary Information:**

The online version contains supplementary material available at 10.1186/s12866-024-03212-9.

## Background

The high mortality and disability rates associated with the prevalence of multi-drug-resistant organisms (MDROs) have attracted widespread attention worldwide [1]. Gram-negative bacteria are important pathogens in the clinic, of which the top five isolation rates in China were *Escherichia coli, Klebsiella pneumoniae, Pseudomonas aeruginosa, Acinetobacter baumannii* and *Enterobacter cloacae* [2]. To date, carbapenems have been recognized as the last resort for clinical treatment of multi-drug resistant gram-negative bacteria [3]. However, carbapenem resistance in gram-negative bacteria has caused a global epidemic that continues to grow. Significantly, we should pay more attention to carbapenem-resistant organisms (CROs), mainly including carbapenem-resistant *Enterobacteriaceae* (CRE), carbapenem-resistant *Acinetobacter baumannii* (CRAB), and carbapenem-resistant *Pseudomonas aeruginosa* (CRPA). The emergence of CROs often limits the choice of antibiotics in the clinic, and the empirical antibiotic therapy does not cover the antimicrobial spectrum of the strain, thus affecting patient prognosis [4]. The China Antibiotic Resistance Surveillance System showed that the resistance rate of gram-negative bacteria in Henan was much higher than the national average in 2021, with the detection rate of CRAB, carbapenem-resistant *Klebsiella pneumoniae* (CRKP), and third-generation cephalosporin-resistant *Klebsiella pneumoniae* being the highest in China [2].

Producing carbapenemase is the most critical mechanism in CROs, especially in *Enterobacterales* [5]. Major carbapenemase genes are often localized on conjugative plasmids, and horizontal transfer of plasmids is a key factor mediating the spread of antimicrobial resistance genes (ARGs) among different strains [6]. The prevalence of CRE in China is attributed to the dissemination of conservative mobile elements carrying *bla*_NDM_ or *bla*_KPC−2_ on conjugative and non-conjugative plasmids [7].

Plasmids are extrachromosomal fragments of DNA that enable rapid adaptation and evolution by transferring genes conferring selective advantages to their hosts. Notably, the term “replicon” can be used to describe any DNA segment that can self-replicate, such as a plasmid. Alternatively, it can refer to specific regions or genes within the DNA that possess the necessary functions to enable replication [8]. Plasmids are typed based on their ability to coexist in the same strain and assigned to different incompatibility groups based on incompatibility (Inc) [9]. Twenty-seven major plasmid incompatibility groups are associated with ARGs in *Enterobacterales*, with IncF, A/C, and X being the most prevalent in carbapenemase production compared with the other Inc groups [10]. Plasmid identification and classification is an essential parameter in current bacterial typing. The most widely used methods are PCR-based replicon typing (PBRT) and degenerate primer MOB typing (DPMT). PBRT targets the replicons on the plasmids, and DPMT targets the relaxase genes. Because of the rapid identification of the dominant replicons, PBRT is the most commonly used technique for plasmid typing in *Enterobacterales* [11].

Plasmids play a key role in uptake expression, horizontal transfer, and rapid evolution of resistance genes [12–14]. Genome sequencing reveals that the plasmid incompatibility group strongly correlates with transfer efficiency [15]. Additionally, plasmids carrying different replicon types often confer different antimicrobial resistance to the host [16]. So, the definition of plasmid replicon types in strains with different antimicrobial phenotypes is indispensable for understanding epidemiological dynamics and making eligible strategies to curb the dissemination of specific plasmids. In this study, we use PBRT to clarify the distribution differences of plasmid replicon types in Henan and explore the association between plasmids and ARGs, aiming to provide a theoretical basis for new ideas in clinical anti-infection treatment.

## Materials and methods

### Strain collection

A retrospective epidemiologic surveillance study of gram-negative strain infection was conducted in a tertiary hospital in Henan. 330 strains from active infections were collected from February to September 2022 during our routine surveillance in this study. All samples were collected using sterile cotton swabs and stored at − 80 °C in brain heart infusion broth with 20% glycerol until use [17]. A VITEK mass spectrometer was used for species identification, and a VITEK-compact 2 automatic microbial system was used for the antimicrobial susceptibility test (AST). *E. coli* ATCC8739 was used as quality control. The antibiotics selected for clinical AST of these 330 strains varied according to the species, patients’ medication history, and individualized treatment plans for patients. The strains were divided into four groups according to the result of AST (Additional file 1): Group A was 93 strains that were fully sensitive to antibiotics, Group B was 53 strains that were resistant to 1–2 kinds of antibiotics, Group C was 91 strains that were resistant to three or more kinds of antibiotics but did not contain carbapenems, and Group D was 93 strains that were resistant to carbapenems. Group D was primarily derived from different strains of the same patient to clarify the possibility of plasmid transmission in different strains.

### Detection of carbapenemase and plasmid replicon typing

All the CROs (Group D) were tested for the presence of the major carbapenemase genes (*bla*_KPC_, *bla*_NDM_, *bla*_OXA−48_, *bla*_IMP_, and *bla*_VIM_) by polymerase chain reaction (PCR) with specific primers and conditions as described previously [18]. To determine the distribution of plasmid incompatibility groups among the four group strains, thirty-three different plasmid replicons, including HI1, HI2, I1-α, I2, X1, X2, X3, X4, M, N, FIA, FIB, W, Y, P1-α, FIC, A/C, T, FII_S_, FII, FII_k,_ FII_Y_, FIB_S_, FIB_K_, L, K1, K2, B/O, R, U, Z, HIB-M, and FIB-M were determined by using PCR-based replicon typing (PBRT) as described previously [11, 19, 20]. The positive PCR amplicons were sequenced and compared with the reported sequences from GenBank by Blast (www.ncbi.nlm.nih.gov/blast/).

### Statistical analysis

The differences in the distribution of plasmid replicons between different groups/species were assessed by Fisher’s exact test or Chi-square test with Yates’ correction using GraphPad Prism8 software (https://www.graphpad.com/). The differences were considered significant when p value was less than 0.05.

## Results

### Sources and characteristics of clinic isolates

**Fig. 1:**
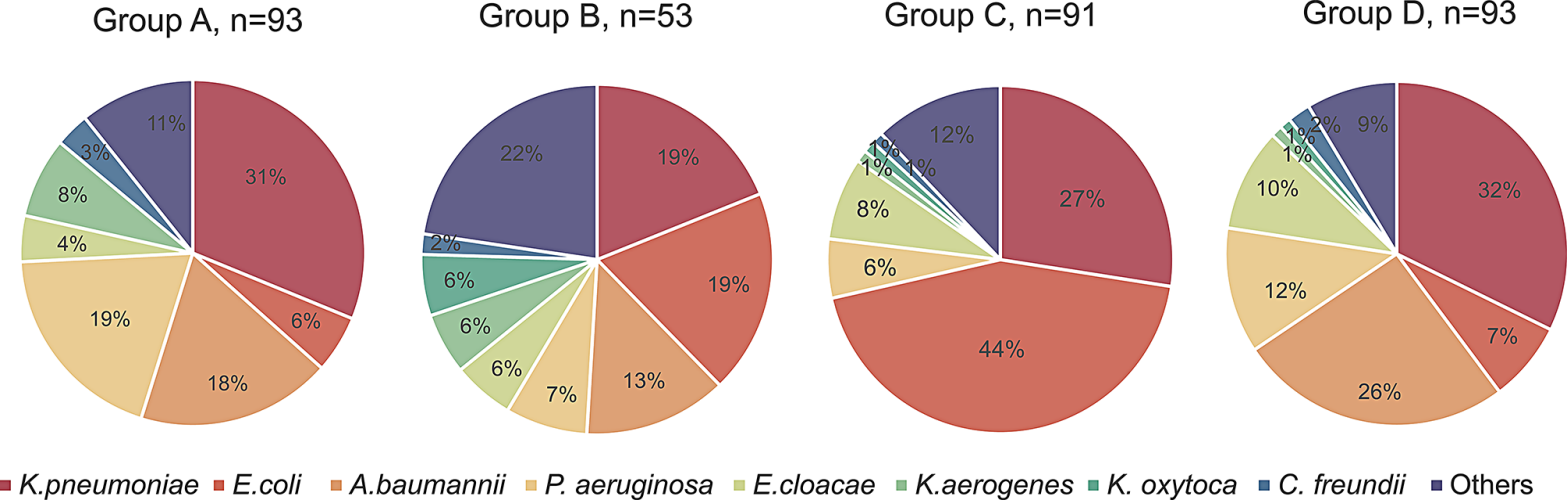
The number of different species

The distribution of strains in each group is shown in Fig. 1. *K. pneumoniae* was the strain with a high clinical isolation rate and was the main component of each group. Compared to the other groups, the clinical isolation rate of *E. coli* in Group C was higher and generally resistant to third-generation cephalosporin and quinolones (Additional file 2). At this time, *A. baumannii* was not collected in Group C. The clinical characteristics of each group are shown in Table 1. In the four groups patients’ age was between 37 and 72 years old. The age distributions in Groups A and B were greater than in Groups C and D. The youngest patient in Group A was eight days. The clinical isolation rate of strains in ICU and urology was higher, among which 45.16% of carbapenem-resistant strains were isolated from ICU, with a more concentrated distribution. In contrast, strains in Groups A and B were distributed in a wide range of clinical departments with outpatients. Specimens of respiratory origin were the main components of this collection.

**Table 1:** Clinical characteristics of infected patients among different groups

Clinical characteristics	Group A No./Total (%)	Group B No./Total (%)	Group C No./Total (%)	Group D No./Total (%)
Male	56/93(60.22%)	31/53(58.49%)	42/91(46.15%)	67/93(72.04%)
Age (years)				
0–18	4/93(4.30%)	4/53(7.55%)	5/91(5.49%)	9/93(9.68%)
19–36	8/93(8.60%)	5/53(9.43%)	3/91(3.30%)	3/93(3.23%)
37–54	36/93(38.71%)	20/53(37.74%)	36/91(39.56%)	30/93(32.26%)
55–72	38/93(40.86%)	19/53(35.85%)	35/91(38.46%)	38/93(40.86%)
73–90	6/93(6.45%)	4/53(7.55%)	12/91(13.19%)	13/93(13.98%)
≥ 90	1/93(1.08%)	1/53(1.89%)	0	0
Department				
Intensive care unit (ICU)	12/93(12.90%)	7/53(13.21%)	12/91(13.19%)	42/93(45.16%)
Urology	14/93(15.05%)	8/53(15.09%)	25/91(27.47%)	4/93(4.30%)
Respiratory Medicine	7/93(7.53%)	1/53(1.89%)	5/91(5.49%)	5/93(5.38%)
Pediatric	2/93(2.15%)	3/53(5.66%)	1/91(1.10%)	3/93(3.23%)
Other	58/93(62.37%)	34/53(64.15%)	48/91(52.75%)	39/93(41.94%)
Source of isolates				
Respiratory tract	48/93(51.61%)	16/53(30.19%)	18/91(19.78%)	47/93(50.54%)
Urinary tract	22/93(23.66%)	13/53(24.53%)	39/91(42.86%)	10/93(10.75%)
Blood	3/93(3.23%)	2/53(3.77%)	5/91(5.49%)	3/93(3.23%)
Other	20/93(21.51%)	22/53(41.51%)	29/91(31.87%)	33/93(35.48%)

### Prevalence of plasmid replicons in four groups and differences in specific species

A total of 21 plasmid replicons were detected (Table 2), and the most common plasmid replicons carried by strains were IncR and IncF-type, including IncFII, IncFIA, and IncFIB plasmid replicons. The number of plasmids carried by strains in the four groups varied widely, indicating that the antimicrobial resistance of the strains was correlated with the number of plasmid replicons, and the number of plasmid replicons increased with the increasing of antimicrobial resistance of the strains (χ2 = 73.983, *p* < 0.001). The number of plasmid replicons in *Enterobacterales* was significantly higher than in the other gram-negative bacteria (Table 3), especially in multi-antimicrobial resistant *Enterobacterales*, where more than half of strains was carrying plasmid replicons (Group C 69.23%, Group D 59.14%).

**Fig. 2:**
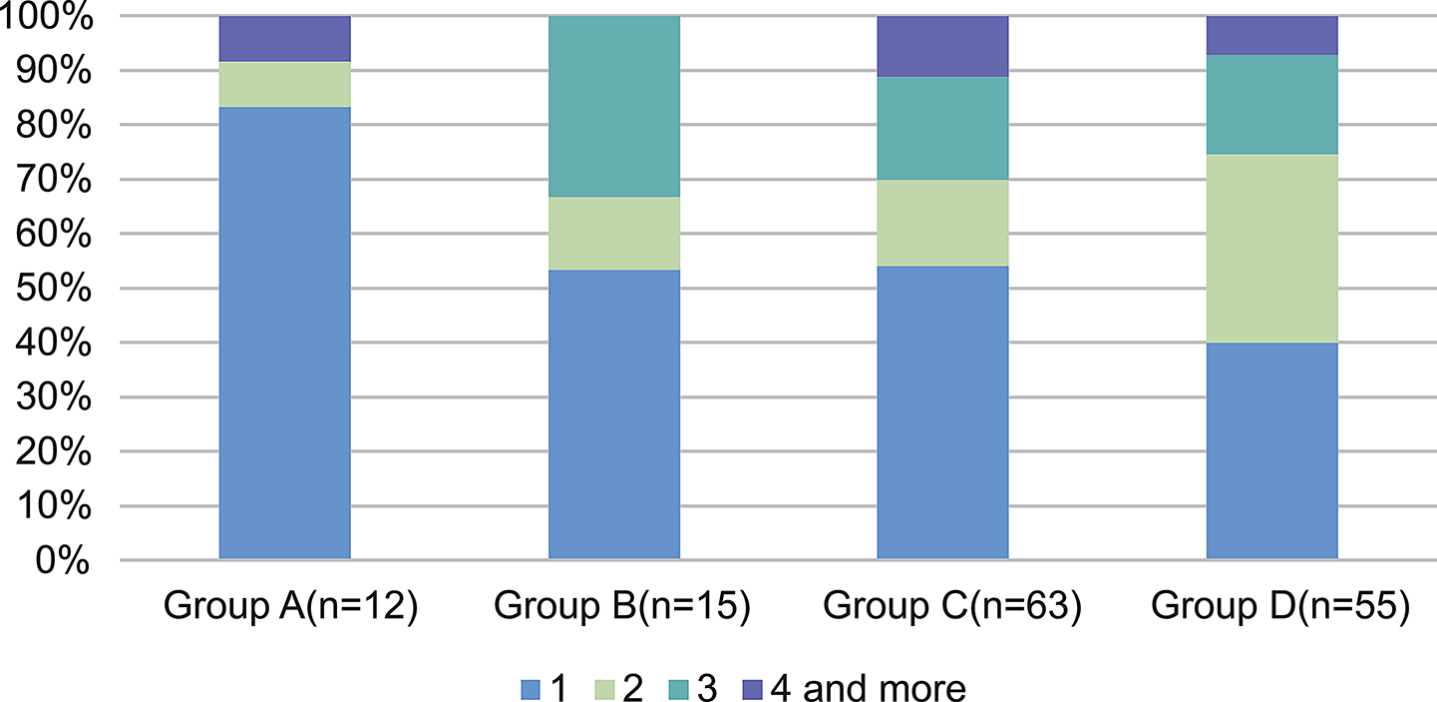
Number of plasmid replicons carried in the same strain among four groups

The same strain carries more than one plasmid replicon type and can carry up to five, like the *E. coli* isolated from wound secretion in Group C. It has five replicon types, IncR, IncFII, IncY, IncFIA, and IncI1α.Groups A and B strains mostly carried 1–2 plasmid types. Notably, one strain of *A. baumannii* isolated from bronchoalveolar lavage fluid of ICU in Group A carried four replicon types, IncFII, IncY, IncFIB, and IncFIA. In contrast, Groups C and D mostly carried two or more plasmid types (Fig. 2).

**Table 2:** The distribution of plasmid replicons among different groups collected at this time

Plasmid replicons	Group A All sensitive strains	Group B Resistance to 1–2 antibiotics	Group C Resistance to 3 or more antibiotics (except carbapenems)	Group D Carbapenem-Resistant Gram-negative bacteria
IncA/C	0	0	1	4
IncFIB	2	6	20	15
IncR	3	4	9	25
IncFIA	1	4	20	5
IncX1	0	0	1	0
IncX2	0	0	1	0
IncX3	0	0	0	13
IncX4	0	0	1	0
IncFII	3	5	21	21
IncY	1	1	7	2
IncI1-α	1	1	10	4
IncT	0	0	0	1
IncFIB-M	0	0	0	3
IncFIIk	1	0	12	2
IncHI2	0	2	2	4
IncHI1	0	0	0	1
IncHIB-M	4	3	1	3
IncN	0	0	0	2
IncI2	0	0	1	0
IncZ	0	1	12	1
IncK1	0	0	1	0

**Table 3:** The distribution of major plasmid replicons among prevalent gram-negative bacteria

Plasmid replicons	*Enterobacterales* (*n* = 237)				Other gram-negative bacteria(*n* = 93)			P value
	*K. pneumoniae(n* = 94)	*E. coli* (*n* = 62)	*E.* *cloacae*(*n* = 23)	*Enterobacter*(*n* = 58)	*A. baumannii*(*n* = 48)	*P. aeruginosa*(*n* = 38)	Other(*n* = 58)	
IncFII(*n* = 50)	13	32	0	1	2	2	0	0.0003
IncFIB(*n* = 43)	7	31	0	2	3	0	0	0.0004
IncR(*n* = 41)	26	2	4	5	4	0	0	0.0048
IncFIA(*n* = 30)	3	23	0	2	2	0	0	0.0048

P value: *Enterobacterales* vs. Other gram-negative bacteria

Due to the limited types of plasmid replicons carried by non-*Enterobacterales*, we only analyzed the differences in prevalent plasmid replicon types in the *Enterobacterales* with high clinical separation rates (Table 4). Among *K. pneumoniae*, IncR plasmid replicons are the most popular. IncFII, IncR, and IncFIIk were associated with MDROs, especially IncFII plasmids, which were only found in Group D, and among the fourteen KPC-CRKP, a total of seven strains carried IncFII plasmid replicons and nine strains carried IncR plasmid replicons. Among *E. coli*, IncFII, IncFIB, and IncFIA had the highest separation rates and were associated with MDROs along with IncI1-α replicon types. Combining the two common clinical strains, the association of IncF-like plasmids with antimicrobial resistance should deserve our extensive attention.

**Table 4:** Differences of prevalent plasmid replicon types in *K. pneumoniae* and *E. coli* with different antimicrobial phenotypes

Plasmid replicons	Group A *K. pneumoniae*/ *E. coli*	Group B *K. pneumoniae*/ *E. coli*	Group C *K. pneumoniae*/ *E. coli*	Group D *K. pneumoniae*/ *E. coli*	P value *K. pneumoniae*/ *E. coli*
IncFII	0/1	0/5	0/21	13/5	0.0006/0.0018
IncFIB	0/1	1/5	0/20	6/5	0.2328/0.0034
IncR	1/1	2/0	6/1	17/0	0.0003/>0.9999
IncFIA	0/0	1/3	0/20	2/0	> 0.9999/0.0014
IncI1-α	0/0	0/0	2/8	1/1	0.2639/0.0094
IncFIIk/IncX3	1/0	0/0	9/0	1/4	0.0233/0.1388

P value: non-MDROs (Group A and B) vs. MDROs (Group C and D)

### Distribution of carbapenemase and plasmid replicons in group D

**Fig. 3:**
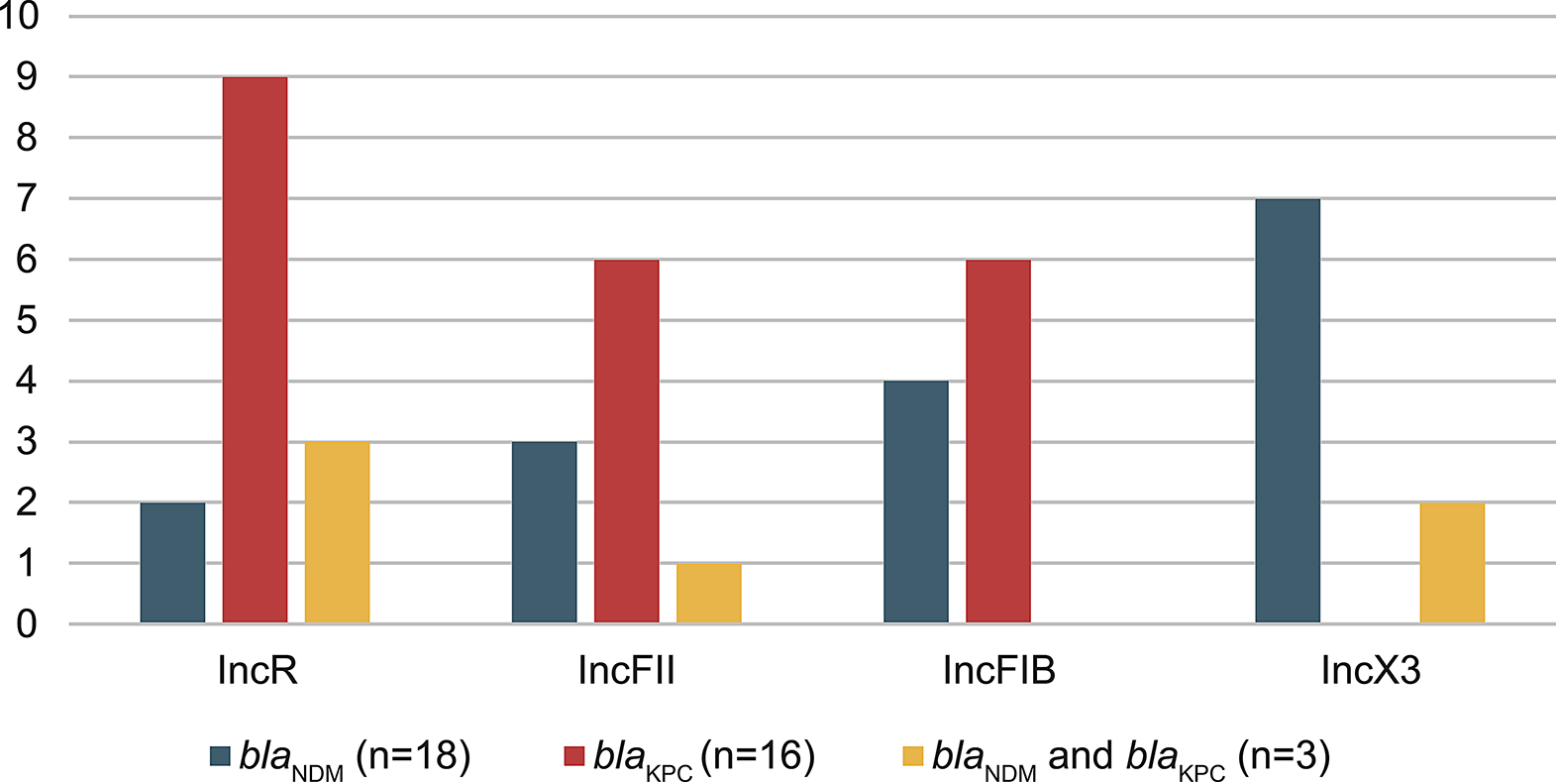
The distribution of prevalent plasmid replicons in strains carrying different resistance determinants

Strains carrying carbapenemase genes localized on the conjugative plasmid are an important cause of carbapenem resistance. Based on this, we tested 93 carbapenemase-resistant strains in Group D for major carbapenemase genes. A total of 37 strains carried carbapenemase genes, 16 carried *bla*_KPC_, 18 carried *bla*_NDM_, and 3 carried *bla*_KPC_ and *bla*_NDM_. 8 strains did not have any plasmid replicon, and 22 strains carried two or more plasmid replicon types, among which the mainstream types were IncR, IncFII, IncFIB, and IncX3. Due to the not straightforward relationship between resistance phenotype and resistance genes, we also selected 73 strains carrying these replicons in other three groups to screen the *bla*_NDM_ and *bla*_KPC_ genes. And the carbapenemase genes tested this time all concentrated in Group D. Figure 3 shows the distribution of plasmid replicon types in strains carrying different antimicrobial resistance genes. In addition, we found a cerebrospinal fluid-derived carbapenem-resistant *Providencia rettgeri*, which carried *bla*_NDM_ and IncT plasmid replicon, and the co-existence of resistance genes and plasmids needs further investigation.

Carbapenemase production is the principal mechanism of carbapenem resistance in CRE, and the carbapenemase genes were usually reported to be located on the plasmid. To determine the association of plasmid incompatibility groups with major carbapenemase genes and to compensate for the lack of data volume in this experiment, we searched the PLSDB database [21] (https://www.ccb.uni-saarland.de/plsdb.) for the common plasmid replicon types described above (Table 5). The data of each plasmid carrying antimicrobial resistance genes is shown in the table below, with specific information on the plasmids in the additional file (Additional file 3).

**Table 5:** Common plasmids carrying major carbapenemase genes in the PLSDB database

Plasmid	bla_KPC_		bla_NDM_			bla_VIM_	bla_IMP_	bla_OXA−48_	Unmentioned/no^a^
	*bla*_KPC−2_	*bla*_KPC−3_	*bla*_NDM−1_	*bla*_NDM−5_	Other				
IncR(*n* = 460)	143	5	11	2	0	11	2	2	83/202
IncFII(*n* = 2209)	1711	7	5	6	2	0	3	0	305/171
IncX3(*n* = 414)	10	5	62	141	44	0	0	53	34/66
IncFIB(*n* = 2579)	39	20	74	24	1	2	23	9	1467/918

^a^ “unmentioned” means the PLSDB database did not record the ARGs in this plasmid, and ”no” indicates that the plasmid does not carry major carbapenemase genes but other ARGs

According to the database, the strains carrying IncR, IncFII, IncX3, and IncFIB replicon-type plasmids are widely distributed, mainly in *K. pneumonia*, *E. coli*, and other *Enterobacterales*. The plasmids often consist of more than one replicon type, except for the IncX3. More than half of the other three types of plasmids were multireplicon, which can often carry more ARGs, putting the clinical treatment in a difficult situation. Interestingly, IncX3 plasmids are smaller than other plasmids, with their size mostly between 30 and 60 kb, carrying limited ARGs. In contrast, the size of the other three types is larger, carrying more ARGs and a broader antimicrobial resistance spectrum. Notably, excluding the information of plasmids not mentioned in the database, 89.86% (1711/1904) of IncFII plasmids carried *bla*_KPC−2_, 65.00% (247/380) of IncX3 plasmids carried *bla*_NDM,_ and its variants and the IncX3 plasmids carried the largest variety of *bla*_NDM_.

A total of 5662 plasmids were collected, of which 3086 plasmids carried two or more plasmid replicon types. However, a plasmid tends to carry only one major carbapenem resistance gene, and we only found four plasmids carrying two major carbapenemase genes in the database (Table 6). Such plasmid tends to be fusion plasmid, which contains not only one replicon. And it further expands the resistance spectrum of strains, which should be worth our attention.

**Table 6:** The same plasmid carries two carbapenemase genes

Accession number	Plasmidfinder	Major carbapenemase genes	Source	Length	Species	Location
NZ_CP065475.1	IncR-IncFIA	*bla*_IMP−4_ *bla*_NDM−1_	Clinical, urine	397,447 bp	*Klebsiella michiganensis*	China
MN661402.1	IncFII-FIA	*bla*_KPC−2_ *bla*_IMP−4_	Unmentioned	377,346 bp	*Klebsiella quasipneumoniae*	Unmentioned
NZ_CP021328.1	IncFII-IncFIB-IncU	*bla*_KPC−2_ *bla*_IMP−4_	Clinical, necrotic tissue	446,611 bp	*Raoultella ornithinolytica*	China
NZ_CP068835.1	IncX3-ColKP3	*bla*_NDM−5_ *bla*_OXA−181_	Clinical	69,764 bp	*Klebsiella pneumoniae*	Netherlands

Among the strains collected in Group D, there were two strains isolated from the same patient (Table 7). The majority of the isolates were one *Enterobacterales*, and the other one was *A. baumannii* or *P. aeruginosa*, which did not carry any plasmid replicons as well as carbapenem resistance genes on them. Both strains of patient 7 carried IncA/C replicons, but the *bla*_NDM_ was not carried by *K. pneumoniae*, suggesting that *bla*_NDM_ is likely to be located on the IncR plasmid, and further experimental validation is needed in this case.

**Table 7:** Characteristics of two strains isolated from the same patient

Patients NO.	Strain number	Species	Plasmid replicons	Major Carbapenemase Genes
P1	D27	*K. pneumoniae*	IncFIB, IncFIA, IncFII, IncR	*bla*_KPC_
	D28	*A. baumannii*	no	no
P2	D30	*K. aerogenes*	IncFIB, IncFIA, IncR	*bla*_KPC_
	D32	*A. baumannii*	no	no
P3	D36	*K. pneumoniae*	IncI1α	*bla*_KPC_
	D37	*A. baumannii*	no	no
P4	D42	*K. pneumoniae*	IncFIA, IncFIB	*bla*_KPC_
	D43	*E. coli*	IncFIB, IncFII, IncFIB-M	*bla*_KPC_
P5	D46	*K. pneumoniae*	IncN, IncHIB-M	no
	D52	*S. marcescens*	no	no
P6	D53	*K. pneumoniae*	IncFII, IncR	*bla*_KPC_
	D54	*A. baumannii*	no	no
P7	D35	*E. cloacae*	IncA/C, IncR	*bla*_NDM_
	D56	*K. pneumoniae*	IncA/C	no
P8	D58	*K. pneumoniae*	IncX3, IncFII, IncR	*bla*_KPC_ *bla*_NDM_
	D72	*A. baumannii*	no	no
P9	D65	*P. aeruginosa*	no	no
	D70	*A. baumannii*	no	no

## Discussion

Plasmids are common mobile genetic elements that can carry a variety of ARGs and promote the rapid spread of resistance in different strains, the study on plasmids is necessary and urgent. This study clarifies the distribution difference of plasmid replicons in gram-negative bacteria with different antimicrobial phenotypes in Henan.

The number of IncF-type, including IncFII, IncFIB, IncFIA, and IncR plasmid replicon types, was the largest. CROs showed a high prevalence of IncR, IncFII, IncX3, and IncFIB replicon types, which is consistent with the study of Zhou et al. [16]. Analysis of the differences in plasmid replicon distribution among clinically common strains with different antimicrobial phenotypes showed that IncF-type plasmids replicons were widely distributed and associated with MDROs. Among *K. pneumoniae*, IncFII and IncR were mainly associated with MDROs, while IncFIA and IncFIB were widely distributed, probably because IncF plasmids can encode several replicons. And mostly multi-replicon plasmids were a combination of IncFII, IncFIA, and IncFIB [22, 23]. *E. coli* was the species that carried the most IncF-type plasmid replicons.

Combined with the PLSDB database, it is clear that IncFII and IncX3 plasmids are the reservoirs of *bla*_KPC−2_ and *bla*_NDM_, respectively. Horizontal transfer mediated by IncFII and IncX3 plasmids plays an essential role in the pandemic expansion of carbapenemase genes. Compared with IncFII plasmid, the IncX3 plasmid is a narrow host range plasmid of *Enterobacteriaceae*, which mainly including *E. coli*, *K. pneumoniae*, *C. freundii*, and *E. cloacae* [24]. IncX3 appears to be the most common type of plasmid carrying *bla*_NDM,_ and it may be a major vehicle in mediating the dissemination of *bla*_NDM_ in East Asia, particularly in China [25]. According to the PLSDB database, *bla*_NDM−5_ is the most prevalent *bla*_NDM_ variant carried by IncX3 plasmids. In Chinese clinical settings, ST167 *E. coli* had close tie to *bla*_NDM−5_ and this ST type may be a potential reservoir relevant to *bla*_NDM−5_ [26]. IncFII plasmid had a broad range host such as *E. coli*, *K. pneumoniae*, *S. enterica*, *C. freundii*, and many other *Enterobacteriaceae.* A close correlation was shown between ST11 KPC-Kp and IncFII-like plasmids, which is the main reason for the transmission of *bla*_KPC−2_ among *K. pneumoniae* ST11 in China [27]. It should be noted that the IncX3 plasmid is not only a reservoir but also an evolution platform of *bla*_NDM_, which carries many variants that have not been reported for other plasmids, such as *bla*_NDM−16b_, *bla*_NDM−17_, *bla*_NDM−20_, *bla*_NDM−21_, and *bla*_NDM−33_ [28–32]. This suggested that the spread of *bla*_NDM_-carrying IncX3 plasmids may be a hotbed for the emergence of novel variants of *bla*_NDM_. And the *bla*_OXA−181_ is mainly located on the IncX3-ColKP3 plasmid, and no other variants have been searched in the database. Although the IncR plasmid carries limited carbapenemase genes, it carries more of the other ARGs. Its conserved backbones include the multidrug-resistant (MDR) regions that can facilitate the integration of antimicrobial resistance genes [33]. IncFIB is also involved in the composition of virulence plasmids and is closely associated with the formation and spread of multidrug-resistant hypervirulent *Klebsiella pneumoniae* (MDR-hvKp) [34, 35].

IncX3, IncT, IncHI1, IncFIB-M, and IncN have not been found in the carbapenem-susceptible strains of this experiment. This may be related to the fact that plasmids are lost when they do not carry resistance genes to reduce the cost of adaptation to bacteria [36]. Interestingly, the IncX family plasmids were all present in *E. coli* of Group C except IncX3. A database search revealed four strains carrying two major carbapenemase genes and localized to the same plasmid. A genetic feature description of a clinically derived strain carrying *bla*_KPC−2_ and *bla*_IMP−4_ was also reported by Dong et al. [37]. This suggests the great potential of fusion plasmids in the integration of resistance genes and the need to be alert to the spread and prevalence of fusion plasmids in the clinical setting.

In the current collection, strains resistant to three or more antibiotics carried a higher number of plasmid replicons. Still, the difference between Groups C and D was not significant, which may be related to the different strain compositions of the two groups and the higher number of *Enterobacterales* strains carried in Group C. The plasmid replicon types of *Enterobacterales* are more numerous and complex than those of other gram-negative bacteria and may also be limited by the experimental method of PBRT [38]. Interestingly, there is a strain of *A. baumannii* carrying four plasmid replicon types among all-sensitive strains, which has the potential to form megaplasmid, and large plasmids are a bridge between the environment and the clinic, with high stability, low fitness cost, and efficient transmission ability to help the dissemination of resistant genes in any environment [39].

Transferability is a significant property of plasmids, and the ability to transfer is certainly correlated with the incompatibility group plasmid replicons. Common plasmid replicons, such as IncF-type, IncI (IncIα, IncI2), IncK, IncB/O, IncZ, IncA/C, IncHI1(temperature-dependent), IncHI2, IncP, IncN, IncX3, IncT, and IncU are conjugative plasmids. However, IncR plasmids are non-conjugative plasmids because of lacking transfer genes [10, 22, 40]. Non-conjugative plasmids with resistance genes could also be co-transferred with a conjugative plasmid [41]. Additionally, resistance genes also have corresponding transposable elements that help ARGs to transfer, such as *bla*_NDM_ with IS*Aba125* and Tn*125*, *bla*_OXA−181_-like genes with IS*Ecp1*, *mcr-1* and *mcr-2* with IS*Apl1* and Tn*6330*, *bla*_SHV_ with IS*26* and Tn*2003* [42]. Further researches on the relationship between plasmid replicons, ARGs, and transposable elements are needed.

In conclusion, as strains become more resistant, the number of plasmid replicons they carry increases. Strains with a broad resistance spectrum often carry more than one plasmid replicon type, and the fusion of multiple replicons may potentially enhance the resistance potential of the strain. There is a correlation between the plasmid replicon type and the resistance genes carried. The high prevalence of IncFII, IncR, and IncFIB plasmid replicons in our study alerts us to the urgency of implementing antimicrobial resistance surveillance, and inhibiting the dissemination and evolution of resistance genes in the form of plasmids is an important way to interrupt the dissemination of resistance. Because of the important role of plasmid in the uptake, transmission, and evolution of ARGs, further research is needed.

### Electronic supplementary material

Below is the link to the electronic supplementary material.

**Supplementary Material 1**: Antimicrobial susceptibility testing of 330 strains

**Supplementary Material 2**: Antimicrobial susceptibility testing of *E. coli* in Group C

**Supplementary Material 3**: The information of plasmid IncR, IncFII, IncFIB, and IncX3 in PLSDB

## Data Availability

The datasets generated and/or analysed during the current study are available in the PLSDB repository, https://www.ccb.uni-saarland.de/plsdb.

## Abbreviations

ARGs: Antimicrobial resistance genes
AST: Antimicrobial susceptibility test
PBRT: PCR-based replicon typing
CROs: Carbapenem-resistant Organisms
MDROs: Multi-Drug-Resistant Organisms
CRE: Carbapenem-resistant *Enterobacteriaceae*
CRAB: Carbapenem-resistant *Acinetobacter baumannii*
CRPA: Carbapenem-resistant *Pseudomonas aeruginosa*
CRKP: Carbapenem-resistant *Klebsiella pneumoniae*
DPMT: Degenerate primer MOB typing
KPC: *Klebsiella pneumoniae* Carbapenemase
PCR: Polymerase chain reaction
MDR-hvKp: Multidrug-resistant hypervirulent *Klebsiella pneumoniae*

## References

[1] Poirel L, Dortet L, Bernabeu S, Nordmann P (2011). Genetic features of bla(NDM-1)-Positive Enterobacteriaceae. Antimicrob Agents Chemother.

[2] Available from: http://www.carss.cn/Report/Details?aId=862.

[3] Armstrong T, Fenn SJ, Hardie KR. JMM Profile: Carbapenems: a broad-spectrum antibiotic. J Med Microbiol. 2021;70(12). 10.1099/jmm.0.001462 PMC8744278 34889726

[4] Wang Z, Qin RR, Huang L, Sun LY (2018). Risk factors for Carbapenem-resistant Klebsiella pneumoniae infection and mortality of Klebsiella pneumoniae infection. Chin Med J (Engl).

[5] Cassini A, Hogberg LD, Plachouras D, Quattrocchi A, Hoxha A, Simonsen GS (2019). Attributable deaths and disability-adjusted life-years caused by infections with antibiotic-resistant bacteria in the EU and the European Economic Area in 2015: a population-level modelling analysis. Lancet Infect Dis.

[6] Gao H, Liu Y, Wang R, Wang Q, Jin L, Wang H (2020). The transferability and evolution of NDM-1 and KPC-2 co-producing Klebsiella pneumoniae from clinical settings. EBioMedicine.

[7] Zhang R, Liu LZ, Zhou HW, Chan EW, Li JP, Fang Y (2017). Nationwide Surveillance of Clinical Carbapenem-resistant Enterobacteriaceae (CRE) strains in China. EBioMedicine.

[8] Carattoli A, Zankari E, García-Fernández A, Voldby Larsen M, Lund O, Villa L (2014). In silico detection and typing of plasmids using PlasmidFinder and plasmid multilocus sequence typing. Antimicrob Agents Chemother.

[9] Novick RP (1987). Plasmid incompatibility. Microbiol Rev.

[10] Kopotsa K, Osei Sekyere J, Mbelle NM (2019). Plasmid evolution in carbapenemase-producing Enterobacteriaceae: a review. Ann N Y Acad Sci.

[11] Villa L, Carattoli A (2020). Plasmid typing and classification. Methods in molecular biology. (Clifton NJ).

[12] Liu ZH, Wang K, Zhang YR, Xia LN, Zhao L, Guo CM (2022). High prevalence and diversity characteristics of Bla(NDM), mcr, and bla(ESBLs) harboring multidrug-resistant Escherichia coli from Chicken, Pig, and cattle in China. Front Cell Infect Microbiol.

[13] Sheppard RJ, Barraclough TG, Jansen VAA (2021). The evolution of plasmid transfer rate in Bacteria and its effect on plasmid persistence. Am Nat.

[14] Ariyoshi T, Aoki K, Kubota H, Sadamasu K, Ishii Y, Tateda K. Molecular characterization of bla(NDM)-Carrying IncX3 plasmids: bla(NDM-16b) likely emerged from a mutation of bla(NDM-5) on IncX3 plasmid. Microbiol Spectr.7. 10.1128/spectrum.01449-22 PMC9430178 35867355

[15] Bethke JH, Davidovich A, Cheng L, Lopatkin AJ, Song WC, Thaden JT (2020). Environmental and genetic determinants of plasmid mobility in pathogenic Escherichia coli. Sci Adv.

[16] Zhou H, Zhang K, Chen W, Chen J, Zheng J, Liu C (2020). Epidemiological characteristics of carbapenem-resistant Enterobacteriaceae collected from 17 hospitals in Nanjing district of China. Antimicrob Resist Infect Control.

[17] Sanderson KE, Zeigler DR (1991). Storing, shipping, and maintaining records on bacterial strains. Methods Enzymol.

[18] Poirel L, Walsh TR, Cuvillier V, Nordmann P (2011). Multiplex PCR for detection of acquired carbapenemase genes. Diagn Microbiol Infect Dis.

[19] Moran RA, Anantham S, Pinyon JL, Hall RM (2015). Plasmids in antibiotic susceptible and antibiotic resistant commensal Escherichia coli from healthy Australian adults. Plasmid.

[20] Rozwandowicz M, Brouwer MS, Zomer AL, Bossers A, Harders F, Mevius DJ et al. Plasmids of distinct IncK lineages show compatible phenotypes. Antimicrob Agents Chemother. 2017;61(3). 10.1128/AAC.01954-16 PMC5328535 28052854

[21] Schmartz GP, Hartung A, Hirsch P, Kern F, Fehlmann T, Müller R (2022). PLSDB: advancing a comprehensive database of bacterial plasmids. Nucleic Acids Res.

[22] Rozwandowicz M, Brouwer MSM, Fischer J, Wagenaar JA, Gonzalez-Zorn B, Guerra B (2018). Plasmids carrying antimicrobial resistance genes in Enterobacteriaceae. J Antimicrob Chemother.

[23] Zurfluh K, Glier M, Hächler H, Stephan R (2015). Replicon typing of plasmids carrying blaCTX-M-15 among Enterobacteriaceae isolated at the environment, livestock and human interface. Sci Total Environ.

[24] Zhu W, Wang X, Qin J, Liang W, Shen Z. Dissemination and Stability of the bla(NDM-5)-Carrying IncX3-Type plasmid among Multiclonal Klebsiella pneumoniae isolates. mSphere. 2020;5(6). 10.1128/mSphere.00917-20 PMC7643832 33148824

[25] Wu W, Feng Y, Tang G, Qiao F, McNally A, Zong Z. NDM Metallo-β-Lactamases and their bacterial producers in Health Care settings. Clin Microbiol Rev. 2019;32(2). 10.1128/CMR.00115-18 PMC6431124 30700432

[26] Li X, Fu Y, Shen M, Huang D, Du X, Hu Q (2018). Dissemination of bla(NDM-5) gene via an IncX3-type plasmid among non-clonal Escherichia coli in China. Antimicrob Resist Infect Control.

[27] Fu P, Tang Y, Li G, Yu L, Wang Y, Jiang X (2019). Pandemic spread of bla((KPC-2)) among Klebsiella pneumoniae ST11 in China is associated with horizontal transfer mediated by IncFII-like plasmids. Int J Antimicrob Agents.

[28] Ariyoshi T, Aoki K, Kubota H, Sadamasu K, Ishii Y, Tateda K (2022). Molecular characterization of bla(NDM)-Carrying IncX3 plasmids: bla(NDM-16b) likely emerged from a mutation of bla(NDM-5) on IncX3 plasmid. Microbiol Spectr.

[29] Liu Z, Wang Y, Walsh TR, Liu D, Shen Z, Zhang R et al. Plasmid-mediated novel bla(NDM-17) gene encoding a carbapenemase with enhanced activity in a sequence type 48 Escherichia coli strain. Antimicrob Agents Chemother. 2017;61(5). 10.1128/AAC.02233-16 PMC5404555 28242668

[30] Liu Z, Li J, Wang X, Liu D, Ke Y, Wang Y (2018). Novel variant of New Delhi Metallo-β-lactamase, NDM-20, in Escherichia coli. Front Microbiol.

[31] Liu L, Feng Y, McNally A, Zong Z (2018). blaNDM-21, a new variant of blaNDM in an Escherichia coli clinical isolate carrying blaCTX-M-55 and rmtB. J Antimicrob Chemother.

[32] Wang T, Zhou Y, Zou C, Zhu Z, Zhu J, Lv J (2021). Identification of a novel bla(NDM) variant, bla(NDM-33,) in an Escherichia coli isolate from Hospital Wastewater in China. mSphere.

[33] Potter RF, D’Souza AW, Dantas G (2016). The rapid spread of carbapenem-resistant Enterobacteriaceae. Drug Resist Updat.

[34] Tian D, Wang M, Zhou Y, Hu D, Ou HY, Jiang X (2021). Genetic diversity and evolution of the virulence plasmids encoding aerobactin and salmochelin in Klebsiella pneumoniae. Virulence.

[35] Musicha P, Msefula CL, Mather AE, Chaguza C, Cain AK, Peno C (2019). Genomic analysis of Klebsiella pneumoniae isolates from Malawi reveals acquisition of multiple ESBL determinants across diverse lineages. J Antimicrob Chemother.

[36] Andersson DI, Hughes D (2010). Antibiotic resistance and its cost: is it possible to reverse resistance?. Nat Rev Microbiol.

[37] Dong H, Liu Z, Wu Z, Zhang T, Xia Z, Zhao Y (2023). Characterization of a conjugative hybrid plasmid coharboring bla(KPC-2) and bla(IMP-4) in a Klebsiella quasipneumoniae clinical isolate. Microbiol Spectr.

[38] Carattoli A (2009). Resistance plasmid families in Enterobacteriaceae. Antimicrob Agents Chemother.

[39] Hall JPJ, Botelho J, Cazares A, Baltrus DA (2022). What makes a megaplasmid?. Philos Trans R Soc Lond B Biol Sci.

[40] Citterio B, Andreoni F, Simoni S, Carloni E, Magnani M, Mangiaterra G (2020). Plasmid replicon typing of antibiotic-resistant Escherichia coli from clams and Marine sediments. Front Microbiol.

[41] Zhai W, Tian Y, Lu M, Zhang M, Song H, Fu Y (2022). Presence of Mobile Tigecycline Resistance Gene tet(X4) in clinical Klebsiella pneumoniae. Microbiol Spectr.

[42] Partridge SR, Kwong SM, Firth N, Jensen SO. Mobile Genetic Elements Associated with Antimicrobial Resistance. Clin Microbiol Rev. 2018;31(4). 10.1128/CMR.00088-17 PMC6148190 30068738

