# Supplementary material for: Difference analysis and characteristics of incompatibility group plasmid replicons in gram-negative bacteria with different antimicrobial phenotypes in Henan, China
Source: BMC Microbiol. 2024 Feb 19;24:64. doi: 10.1186/s12866-024-03212-9 (PMC10875880; doi:10.1186/s12866-024-03212-9)
Supplement: Supplementary file 1 — Supplementary Material 1: Antimicrobial susceptibility testing of 330 strains [file 12866_2024_3212_MOESM1_ESM.docx]

Table 1 Antimicrobial susceptibility testing of 330 strains

| NO. | DOX | COL | TZP | TCC | CSL | SAM | CAZ | FEP | MEM | IPM | TOB | AMK | LVX | CIP | SXT | MNO | TGC | CRO |  |  |  |  |
| --- | --- | --- | --- | --- | --- | --- | --- | --- | --- | --- | --- | --- | --- | --- | --- | --- | --- | --- | --- | --- | --- | --- |
| A5 | ≤0.5 | ≤0.5 | ≤4 | ≤8 | ≤8 | 22 | 2 | 2 | ≤0.25 | ≤0.25 | ≤1 | ≤2 | ≤0.12 | ≤0.25 | ≤1/19 | ≤1 | ≤0.5 | —— |  |  |  |  |
| A9 | ≤0.5 | ≤0.5 | ≤4 | ≤8 | ≤8 | 25 | 2 | 2 | ≤0.25 | ≤0.25 | ≤1 | 4 | ≤0.12 | ≤0.25 | ≤1/19 | ≤1 | ≤0.5 | —— |  |  |  |  |
| A10 | ≤0.5 | 2 | —— | ≤8 | ≤8 | —— | 2 | —— | ≤0.25 | ≤0.25 | ≤1 | 4 | ≤0.12 | ≤0.25 | ≤1/19 | ≤1 | ≤0.5 | —— |  |  |  |  |
| A11 | ≤0.5 | 1 | ≤4 | ≤8 | ≤8 | —— | 1 | 0.5 | ≤0.25 | ≤0.25 | ≤1 | ≤2 | ≤0.12 | ≤0.25 | ≤1/19 | ≤1 | ≤0.5 | —— |  |  |  |  |
| A12 | ≤0.5 | ≤0.5 | ≤4 | ≤8 | ≤8 | 23 | 2 | 2 | ≤0.25 | ≤0.25 | ≤1 | ≤2 | ≤0.12 | ≤0.25 | ≤1/19 | 2 | ≤0.5 | —— |  |  |  |  |
| A25 | ≤0.5 | ≤0.5 | ≤4 | ≤8 | ≤8 | 26 | 2 | 2 | ≤0.25 | ≤0.25 | ≤1 | ≤2 | ≤0.12 | ≤0.25 | ≤1/19 | ≤1 | ≤0.5 | —— |  |  |  |  |
| A35 | ≤0.5 | ≤0.5 | ≤4 | ≤8 | ≤8 | 23 | 2 | 2 | ≤0.25 | ≤0.25 | ≤1 | ≤2 | ≤0.12 | ≤0.25 | ≤1/19 | ≤1 | ≤0.5 | —— |  |  |  |  |
| A36 | ≤0.5 | ≤0.5 | 8 | ≤8 | ≤8 | 24 | 2 | 2 | ≤0.25 | ≤0.25 | ≤1 | ≤2 | ≤0.12 | ≤0.25 | ≤1/19 | 2 | ≤0.5 | —— |  |  |  |  |
| A52 | ≤0.5 | ≤0.5 | ≤4 | ≤8 | ≤8 | 26 | 2 | 2 | ≤0.25 | ≤0.25 | ≤1 | ≤2 | ≤0.12 | ≤0.25 | ≤1/19 | ≤1 | ≤0.5 | —— |  |  |  |  |
| A56 | ≤0.5 | ≤0.5 | ≤4 | ≤8 | ≤8 | 20 | 2 | 2 | ≤0.25 | ≤0.25 | ≤1 | ≤2 | ≤0.12 | ≤0.25 | ≤1/19 | 4 | ≤0.5 | —— |  |  |  |  |
| A61 | ≤0.5 | ≤0.5 | ≤4 | ≤8 | ≤8 | 16 | 2 | 2 | ≤0.25 | ≤0.25 | ≤1 | ≤2 | ≤0.12 | ≤0.25 | ≤1/19 | ≤1 | ≤0.5 | —— |  |  |  |  |
| A68 | ≤0.5 | ≤0.5 | ≤4 | ≤8 | ≤8 | 25 | 2 | 2 | ≤0.25 | ≤0.25 | ≤1 | ≤2 | ≤0.12 | ≤0.25 | ≤1/19 | ≤1 | ≤0.5 | —— |  |  |  |  |
| A78 | ≤0.5 | 1 | ≤4 | ≤8 | ≤8 | 21 | 2 | —— | ≤0.25 | ≤0.25 | ≤1 | ≤2 | ≤0.12 | ≤0.25 | ≤1/19 | ≤1 | ≤0.5 | —— |  |  |  |  |
| A79 | ≤0.5 | ≤0.5 | ≤4 | ≤8 | ≤8 | 16 | 2 | 2 | ≤0.25 | ≤0.25 | ≤1 | ≤2 | ≤0.12 | ≤0.25 | ≤1/19 | ≤1 | ≤0.5 | —— |  |  |  |  |
| A83 | ≤0.5 | ≤0.5 | ≤4 | ≤8 | ≤8 | 26 | 2 | 2 | ≤0.25 | ≤0.25 | ≤1 | ≤2 | ≤0.12 | ≤0.25 | ≤1/19 | ≤1 | ≤0.5 | —— |  |  |  |  |
| A84 | ≤0.5 | 2 | ≤4 | ≤8 | ≤8 | 24 | 2 | 4 | ≤0.25 | ≤0.25 | ≤1 | ≤2 | ≤0.12 | ≤0.25 | ≤1/19 | ≤1 | ≤0.5 | —— |  |  |  |  |
| A85 | ≤0.5 | ≤0.5 | 8 | ≤8 | ≤8 | 25 | 2 | 1 | ≤0.25 | ≤0.25 | ≤1 | ≤2 | ≤0.12 | ≤0.25 | ≤1/19 | ≤1 | ≤0.5 | —— |  |  |  |  |
| A88 | ≤0.5 | ≤0.5 | ≤4 | ≤8 | ≤8 | 26 | 2 | 2 | ≤0.25 | ≤0.25 | ≤1 | ≤2 | ≤0.12 | ≤0.25 | ≤1/19 | ≤1 | ≤0.5 | —— |  |  |  |  |
| A90 | ≤0.5 | ≤0.5 | ≤4 | ≤8 | ≤8 | 26 | 2 | 2 | ≤0.25 | ≤0.25 | ≤1 | ≤2 | ≤0.12 | ≤0.25 | ≤1/19 | ≤1 | ≤0.5 | —— |  |  |  |  |
| A91 | ≤0.5 | ≤0.5 | ≤4 | ≤8 | ≤8 | 26 | 2 | 2 | ≤0.25 | ≤0.25 | ≤1 | ≤2 | ≤0.12 | ≤0.25 | ≤1/19 | ≤1 | ≤0.5 | —— |  |  |  |  |
| B21 | ≤0.5 | ≤0.5 | 8 | ≤8 | ≤8 | 20 | 2 | 2 | ≤0.25 | ≤0.25 | ≤1 | ≤2 | ≤0.12 | ≤0.25 | ≤1/19 | ≤1 | ≤0.5 | 13 |  |  |  |  |
| B30 | ≤0.5 | ≤0.5 | ≥128 | ≤8 | ≤8 | 18 | 18 | 8 | 0.5 | ≤0.25 | ≤1 | ≤2 | ≤0.12 | ≤0.25 | ≤1/19 | ≤1 | ≤0.5 | —— |  |  |  |  |
| B33 | ≤0.5 | ≤0.5 | ≥128 | ≤8 | ≤8 | 21 | 8 | 4 | ≤0.25 | ≤0.26 | ≤1 | ≤2 | ≤0.12 | ≤0.25 | ≤1/19 | ≤1 | ≤0.5 | —— |  |  |  |  |
| B34 | ≥16 | ≤0.5 | 8 | ≤8 | ≤8 | 26 | 2 | 4 | ≤0.25 | ≤0.27 | ≤1 | ≤2 | ≤0.12 | ≤0.25 | 8/152 | ≤1 | ≤0.5 | —— |  |  |  |  |
| B38 | ≤0.5 | ≤0.5 | ≤4 | ≤8 | ≤8 | 22 | 2 | 2 | ≤0.25 | ≤0.28 | ≤1 | ≤2 | ≤0.12 | ≤0.25 | ≤1/19 | ≤1 | ≤0.5 | 6 |  |  |  |  |
| B45 | ≤0.5 | ≤0.5 | ≤4 | ≤8 | ≤8 | 6 | 2 | 2 | ≤0.25 | ≤0.29 | ≤1 | ≤2 | ≤0.12 | ≤0.25 | ≤1/19 | ≤1 | ≤0.5 | 6 |  |  |  |  |
| B58 | ≤0.5 | ≤0.5 | ≥128 | ≤8 | ≤8 | 24 | 8 | 4 | ≤0.25 | ≤0.30 | ≤1 | ≤2 | ≤0.12 | ≤0.25 | ≤1/19 | ≤1 | ≤0.5 | —— |  |  |  |  |
| D4 | ≥16 | ≤0.5 | ≥128 | ≥128 | 16 | 6 | ≥64 | ≥32 | ≥16 | ≥16 | ≥16 | 64 | ≥8 | ≥4 | ≤1/19 | 8 | 2 | —— |  |  |  |  |
| D25 | ≥16 | ≤0.5 | ≥128 | ≥128 | ≥64 | 6 | ≥64 | ≥32 | ≥16 | ≥16 | ≤1 | ≤2 | 4 | ≥4 | ≤1/19 | 4 | 2 | —— |  |  |  |  |
| D28 | 2 | ≤0.5 | ≥128 | ≥128 | ≥64 | 6 | ≥64 | ≥32 | ≥16 | ≥16 | ≥16 | ≥34 | ≥8 | ≥4 | ≥16/304 | 2 | 2 | —— |  |  |  |  |
| D32 | ≥16 | ≤0.5 | ≥128 | ≥128 | ≥64 | 6 | ≥64 | ≥32 | ≥16 | ≥16 | ≥16 | ≥34 | ≥8 | ≥4 | ≥16/304 | 8 | 2 | —— |  |  |  |  |
| D37 | ≥16 | ≤0.5 | ≥128 | ≥128 | ≥64 | 6 | ≥64 | ≥32 | ≥16 | ≥16 | ≥16 | 64 | 4 | ≥4 | ≤1/19 | 4 | 2 | —— |  |  |  |  |
| D54 | ≥16 | ≤0.5 | ≥128 | ≥128 | 16 | 6 | ≥64 | ≥32 | ≥16 | ≥16 | 8 | ≤2 | ≥8 | ≥4 | ≥16/304 | 4 | 2 | —— |  |  |  |  |
| D55 | ≥16 | 2 | ≥128 | ≥128 | 32 | 6 | ≥64 | ≥32 | ≥16 | ≥16 | ≤1 | 4 | ≥8 | ≥4 | ≤1/19 | 4 | 2 | —— |  |  |  |  |
| D60 | ≥16 | ≤0.5 | ≥128 | ≥128 | 32 | 6 | ≥64 | 16 | ≥16 | ≥16 | ≥16 | 64 | 4 | ≥4 | ≤1/19 | 4 | 2 | —— |  |  |  |  |
| D61 | ≥16 | ≤0.5 | ≥128 | ≥128 | ≥64 | 6 | ≥64 | ≥32 | ≥16 | ≥16 | ≥16 | ≥64 | ≥8 | ≥4 | ≥16/304 | 4 | 2 | —— |  |  |  |  |
| D63 | ≥16 | ≤0.5 | ≥128 | ≥128 | ≥64 | 6 | ≥64 | ≥32 | ≥16 | ≥16 | ≥16 | ≥64 | ≥8 | ≥4 | ≥16/304 | 4 | 2 | —— |  |  |  |  |
| D67 | ≥16 | ≤0.5 | ≥128 | ≥128 | ≥64 | 6 | ≥64 | ≥32 | ≥16 | ≥16 | ≥16 | 64 | ≥8 | ≥4 | ≥16/304 | 4 | 2 | —— |  |  |  |  |
| D68 | 2 | ≤0.5 | ≥128 | ≥128 | ≥64 | 6 | ≥64 | ≥32 | ≥16 | ≥16 | ≥16 | 64 | ≥8 | ≥4 | ≥16/304 | 2 | 2 | —— |  |  |  |  |
| D70 | ≥16 | ≤0.5 | ≥128 | ≥128 | ≥64 | 6 | ≥64 | ≥32 | ≥16 | ≥16 | ≥16 | 64 | ≥8 | ≥4 | ≤1/19 | 8 | 2 | —— |  |  |  |  |
| D72 | ≥16 | ≤0.5 | ≥128 | ≥128 | ≥64 | 6 | ≥64 | ≥32 | ≥16 | ≥16 | 8 | ≤2 | ≥8 | ≥4 | ≥16/304 | 4 | 2 | —— |  |  |  |  |
| D75 | ≥16 | ≤0.5 | ≥128 | ≥128 | 32 | 6 | ≥64 | ≥32 | ≥16 | ≥16 | ≥16 | 64 | ≥8 | ≥4 | ≤1/19 | 4 | 2 | —— |  |  |  |  |
| D77 | ≥16 | ≤0.5 | ≥128 | ≥128 | ≥64 | 6 | ≥64 | ≥32 | ≥16 | ≥16 | ≥16 | 4 | 4 | ≥4 | ≥16/304 | 4 | 2 | —— |  |  |  |  |
| D79 | ≥16 | ≤0.5 | ≥128 | ≥128 | ≥64 | 6 | ≥64 | ≥32 | ≥16 | ≥16 | ≥16 | 64 | ≥8 | ≥4 | ≥16/304 | 4 | 2 | —— |  |  |  |  |
| D82 | ≥16 | ≤0.5 | ≥128 | ≥128 | 32 | 6 | ≥64 | ≥32 | ≥16 | ≥16 | ≥16 | 64 | ≥8 | ≥4 | ≤1/19 | 4 | 2 | —— |  |  |  |  |
| D83 | ≥16 | ≤0.5 | ≥128 | ≥128 | ≥64 | 6 | ≥64 | ≥32 | ≥16 | ≥16 | ≥16 | 64 | ≥8 | ≥4 | ≥16/304 | 4 | 2 | —— |  |  |  |  |
| D89 | ≥16 | ≤0.5 | ≥128 | ≥128 | ≥64 | 6 | ≥64 | ≥32 | ≥16 | ≥16 | ≥16 | ≥64 | ≥8 | ≥4 | ≥16/304 | 4 | 1 | —— |  |  |  |  |
| D95 | 1 | ≤0.5 | ≥128 | ≥128 | ≥64 | 6 | ≥64 | ≥32 | ≥16 | ≥16 | ≤1 | ≤2 | ≥8 | ≥4 | 4/76 | ≤1 | 2 | —— |  |  |  |  |
| D96 | ≥16 | ≤0.5 | ≥128 | ≥128 | ≥64 | 6 | ≥64 | ≥32 | ≥16 | ≥16 | ≥16 | 64 | ≥8 | ≥4 | ≤1/19 | 4 | 2 | —— |  |  |  |  |
| D97 | ≥16 | ≤0.5 | ≥128 | ≥128 | ≥64 | 6 | ≥64 | ≥32 | ≥16 | ≥16 | ≥16 | 64 | ≥8 | ≥4 | ≤1/19 | 4 | 2 | —— |  |  |  |  |
| D101 | ≤0.5 | ≤0.5 | ≥128 | ≥128 | ≥64 | 21 | 2 | 2 | ≥16 | ≥16 | ≤1 | ≤2 | ≤0.2 | ≤0.25 | ≤1/19 | 1 | ≤0.5 | —— |  |  |  |  |
| NO. | PIP | TZP | CZO | CAZ | FZP | ATM | AMK | IPM | MEM | GEN | TOB | CIP | LVX |  |  |  |  |  |  |  |  |  |
| A1 | ≤4 | ≤4 | —— | 4 | 2 | 4 | ≤2 | ≤1 | ≤0.25 | ≤1 | ≤1 | ≤0.25 | 0.5 |  |  |  |  |  |  |  |  |  |
| A3 | ≤4 | ≤4 | —— | 2 | ≤1 | 4 | ≤2 | ≤1 | ≤0.25 | ≤1 | ≤1 | ≤0.25 | 0.5 |  |  |  |  |  |  |  |  |  |
| A6 | ≤4 | ≤4 | —— | 2 | 4 | 2 | 4 | 2 | ≤0.25 | 4 | ≤1 | ≤0.25 | 1 |  |  |  |  |  |  |  |  |  |
| A7 | ≤4 | ≤4 | —— | 2 | 4 | 4 | 8 | 2 | ≤0.25 | 4 | ≤1 | ≤0.25 | 1 |  |  |  |  |  |  |  |  |  |
| A8 | ≤4 | ≤4 | —— | 4 | 2 | 4 | ≤2 | 2 | ≤0.25 | ≤1 | ≤1 | ≤0.25 | 0.5 |  |  |  |  |  |  |  |  |  |
| A20 | ≤4 | ≤4 | —— | ≤1 | ≤1 | 2 | ≤2 | 2 | ≤0.25 | ≤1 | ≤1 | ≤0.25 | ≤0.25 |  |  |  |  |  |  |  |  |  |
| A38 | 8 | ≤4 | —— | 4 | 4 | 8 | ≤2 | 2 | ≤0.25 | ≤1 | ≤1 | ≤0.25 | 0.5 |  |  |  |  |  |  |  |  |  |
| A40 | ≤4 | ≤4 | —— | 4 | 2 | 4 | ≤2 | 2 | ≤0.25 | 2 | ≤1 | ≤0.25 | 0.5 |  |  |  |  |  |  |  |  |  |
| A41 | 8 | 8 | —— | 4 | 4 | 8 | ≤2 | 2 | 1 | 2 | ≤1 | ≤0.25 | 0.5 |  |  |  |  |  |  |  |  |  |
| A42 | ≤4 | ≤4 | —— | 4 | 4 | 4 | ≤2 | 2 | 1 | ≤1 | ≤1 | ≤0.25 | 0.5 |  |  |  |  |  |  |  |  |  |
| B23 | 16 | 16 | —— | 4 | 4 | 32 | ≤2 | ≤1 | 1 | ≤1 | ≤1 | ≤0.25 | 0.5 |  |  |  |  |  |  |  |  |  |
| B24 | 16 | 16 | —— | 4 | 8 | ≥64 | ≤2 | 2 | 1 | ≤1 | ≤1 | 0.5 | 1 |  |  |  |  |  |  |  |  |  |
| B35 | 8 | 8 | —— | 2 | 2 | 16 | ≤2 | ≤1 | 1 | ≤1 | ≤1 | 2 | 4 |  |  |  |  |  |  |  |  |  |
| D65 | ≥128 | 64 | —— | ≥64 | 32 | 16 | 4 | ≥16 | ≥16 | ≤1 | ≤1 | ≤0.25 | 0.5 |  |  |  |  |  |  |  |  |  |
| D78 | ≤4 | ≤4 | —— | 4 | 2 | 4 | ≤2 | 8 | 8 | ≤1 | ≤1 | ≤0.25 | 0.5 |  |  |  |  |  |  |  |  |  |
| D99 | 8 | 8 | —— | 4 | 2 | 16 | ≤2 | 2 | 8 | ≤1 | ≤1 | ≤0.25 | ≤0.25 |  |  |  |  |  |  |  |  |  |
| D100 | ≥128 | ≥128 | —— | ≥64 | ≥64 | 2 | 4 | ≥16 | ≥16 | ≥16 | ≥16 | ≥4 | ≥8 |  |  |  |  |  |  |  |  |  |
| NO. | COL | TCC | TZP | CSL | CAZ | FEP | ATM | IPM | MEM | AMK | TOB |  |  |  |  |  |  |  |  |  |  |  |
| A22 | ≤0.5 | 16 | ≤4 | ≤8 | 2 | 2 | 4 | 2 | 0.5 | ≤2 | ≤1 |  |  |  |  |  |  |  |  |  |  |  |
| A29 | ≤0.5 | 16 | ≤4 | ≤8 | 2 | 4 | 4 | ≤0.25 | ≤0.25 | ≤2 | ≤1 |  |  |  |  |  |  |  |  |  |  |  |
| A30 | ≤0.5 | 16 | ≤4 | ≤8 | 2 | 2 | 4 | 1 | ≤0.25 | 4 | ≤1 |  |  |  |  |  |  |  |  |  |  |  |
| A32 | ≤0.5 | 16 | ≤4 | ≤8 | 2 | 2 | 4 | 1 | ≤0.25 | ≤2 | ≤1 |  |  |  |  |  |  |  |  |  |  |  |
| A34 | ≤0.5 | 16 | ≤4 | ≤8 | 2 | 2 | 2 | 1 | ≤0.25 | ≤2 | ≤1 |  |  |  |  |  |  |  |  |  |  |  |
| A54 | ≤0.5 | 16 | ≤4 | ≤8 | 2 | 2 | 4 | 1 | ≤0.25 | ≤2 | ≤1 |  |  |  |  |  |  |  |  |  |  |  |
| A64 | 2 | 16 | ≤4 | ≤8 | 2 | 2 | 4 | 1 | ≤0.25 | ≤2 | ≤1 |  |  |  |  |  |  |  |  |  |  |  |
| A94 | ≤0.5 | 16 | ≤4 | ≤8 | 2 | 8 | 2 | 2 | ≤0.25 | 4 | ≤1 |  |  |  |  |  |  |  |  |  |  |  |
| B22 | ≤0.5 | 64 | ≥128 | 16 | 8 | 8 | 2 | 2 | 1 | 4 | ≤1 |  |  |  |  |  |  |  |  |  |  |  |
| C25 | ≤0.5 | ≥128 | ≥128 | ≥64 | 2 | 8 | 16 | 2 | 2 | 16 | ≥16 |  |  |  |  |  |  |  |  |  |  |  |
| C53 | ≤0.5 | ≥128 | ≥128 | ≥64 | 16 | 16 | 1 | 1 | ≤2 | —— | ≤1 |  |  |  |  |  |  |  |  |  |  |  |
| C86 | 1 | ≥128 | ≥128 | ≥64 | 32 | 8 | ≥64 | 1 | 1 | ≤2 | ≤1 |  |  |  |  |  |  |  |  |  |  |  |
| C87 | ≤0.5 | ≥128 | ≥128 | ≥64 | ≥64 | ≥32 | ≥64 | 2 | 1 | 4 | ≤1 |  |  |  |  |  |  |  |  |  |  |  |
| C88 | ≥16 | 16 | ≤4 | 8 | 2 | ≥32 | ≥64 | 1 | ≤0.25 | ≤2 | 2 |  |  |  |  |  |  |  |  |  |  |  |
| D11 | ≤0.5 | ≥128 | ≥128 | ≥64 | 32 | 16 | ≥64 | ≥16 | ≥16 | ≤2 | ≤1 |  |  |  |  |  |  |  |  |  |  |  |
| D21 | ≤0.5 | ≥128 | 8 | 16 | 2 | 4 | 16 | ≥16 | ≥16 | ≤2 | ≤1 |  |  |  |  |  |  |  |  |  |  |  |
| D41 | ≤0.5 | 16 | 8 | ≤8 | 2 | 2 | 4 | ≥16 | 4 | ≤2 | ≤1 |  |  |  |  |  |  |  |  |  |  |  |
| D48 | ≤0.5 | ≥128 | 64 | 32 | ≥64 | 4 | 16 | ≥16 | 4 | ≤2 | ≤1 |  |  |  |  |  |  |  |  |  |  |  |
| D57 | ≤0.5 | ≥128 | ≥128 | ≥64 | ≥64 | 16 | ≥64 | ≥16 | ≥16 | ≤2 | ≤1 |  |  |  |  |  |  |  |  |  |  |  |
| D59 | ≤0.5 | ≥128 | ≥128 | ≥64 | 8 | 4 | 2 | ≥16 | ≥16 | ≥64 | ≥16 |  |  |  |  |  |  |  |  |  |  |  |
| D86 | ≤0.5 | ≥128 | ≥128 | ≥64 | 2 | 2 | 8 | ≥16 | 4 | ≤2 | ≤1 |  |  |  |  |  |  |  |  |  |  |  |
| NO. | DOX | AMP | COL | TCC | CSL | TZP | SAM | CZO | CXM | CAZ | CRO | FEP | ATM | IPM | MEM | AMK | TOB | CIP | LVX | SXT | MNO | TGC |
| A17 | 1 | —— | ≤0.5 | ≤8 | ≤8 | ≤4 | 20 | 25 | 22 | ≤0.12 | 27 | ≤0.12 | ≤1 | ≤0.25 | ≤0.25 | ≤2 | ≤1 | ≤0.25 | ≤0.12 | ≤1/19 | 4 | 1 |
| A18 | 1 | —— | ≤0.5 | ≤8 | ≤8 | ≤4 | 16 | 23 | 21 | ≤0.12 | 29 | ≤0.12 | ≤1 | ≤0.25 | ≤0.25 | ≤2 | ≤1 | ≤0.25 | ≤0.12 | ≤1/19 | 2 | ≤0.5 |
| A19 | 1 | —— | ≤0.5 | ≤8 | ≤8 | ≤4 | 24 | 23 | 25 | ≤0.12 | 29 | ≤0.12 | ≤1 | ≤0.25 | ≤0.25 | ≤2 | ≤1 | ≤0.25 | ≤0.12 | ≤1/19 | 2 | ≤0.5 |
| A26 | 1 | —— | ≤0.5 | ≤8 | ≤8 | ≤4 | 18 | 23 | 21 | ≤0.12 | 30 | ≤0.12 | ≤1 | ≤0.25 | ≤0.25 | ≤2 | ≤1 | ≤0.25 | ≤0.12 | ≤1/19 | 2 | ≤0.5 |
| A28 | 1 | —— | ≤0.5 | ≤8 | ≤8 | ≤4 | 20 | 23 | 21 | 0.25 | 26 | ≤0.12 | ≤1 | ≤0.25 | ≤0.25 | ≤2 | ≤1 | ≤0.25 | ≤0.12 | ≤1/19 | 2 | ≤0.5 |
| A31 | 1 | —— | ≤0.5 | ≤8 | ≤8 | ≤4 | 16 | 25 | —— | ≤0.12 | 25 | ≤0.12 | ≤1 | ≤0.25 | ≤0.25 | ≤2 | ≤1 | ≤0.25 | ≤0.12 | ≤1/19 | ≤1 | ≤0.5 |
| A39 | 1 | —— | ≤0.5 | ≤8 | ≤8 | ≤4 | 17 | 23 | 22 | ≤0.12 | 26 | ≤0.12 | ≤1 | ≤0.25 | ≤0.25 | ≤2 | ≤1 | ≤0.25 | ≤0.12 | ≤1/19 | 2 | ≤0.5 |
| A44 | 2 | —— | ≤0.5 | ≤8 | ≤8 | ≤4 | 18 | 26 | 22 | 0.25 | 30 | ≤0.12 | ≤1 | 1 | ≤0.25 | ≤2 | ≤1 | ≤0.25 | ≤0.12 | ≤1/19 | 4 | 1 |
| A45 | 1 | —— | ≤0.5 | ≤8 | ≤8 | ≤4 | 19 | 26 | 24 | 0.25 | 30 | ≤0.12 | 2 | 1 | 0.5 | ≤2 | ≤1 | ≤0.25 | ≤0.12 | ≤1/19 | 4 | 1 |
| A46 | 1 | —— | ≤0.5 | ≤8 | ≤8 | ≤4 | 17 | 23 | 20 | ≤0.12 | 30 | ≤0.12 | ≤1 | ≤0.25 | ≤0.25 | ≤2 | ≤1 | ≤0.25 | ≤0.12 | ≤1/19 | ≤1 | ≤0.5 |
| A49 | 1 | —— | ≤0.5 | ≤8 | ≤8 | ≤4 | 16 | 23 | —— | ≤0.12 | —— | ≤0.12 | ≤1 | ≤0.25 | ≤0.25 | ≤2 | ≤1 | ≤0.25 | ≤0.12 | ≤1/19 | ≤1 | ≤0.5 |
| A50 | 1 | —— | ≤0.5 | ≤8 | ≤8 | ≤4 | 18 | 23 | 20 | ≤0.12 | 23 | ≤0.12 | ≤1 | ≤0.25 | ≤0.25 | ≤2 | ≤1 | ≤0.25 | ≤0.12 | ≤1/19 | 2 | ≤0.5 |
| A51 | 1 | —— | ≤0.5 | ≤8 | ≤8 | ≤4 | 19 | 23 | 21 | ≤0.12 | 25 | ≤0.12 | ≤1 | ≤0.25 | ≤0.25 | ≤2 | ≤1 | ≤0.25 | ≤0.12 | ≤1/19 | 2 | ≤0.5 |
| A59 | 1 | —— | ≤0.5 | ≤8 | ≤8 | ≤4 | 18 | 23 | —— | ≤0.12 | 30 | ≤0.12 | ≤1 | 1 | ≤0.25 | ≤2 | ≤1 | ≤0.25 | ≤0.12 | ≤1/19 | ≤1 | ≤0.5 |
| A62 | 1 | —— | ≤0.5 | ≤8 | ≤8 | ≤4 | 20 | 24 | 20 | ≤0.12 | 26 | ≤0.12 | ≤1 | ≤0.25 | ≤0.25 | ≤2 | ≤1 | ≤0.25 | ≤0.12 | ≤1/19 | ≤1 | ≤0.5 |
| A63 | 1 | —— | ≤0.5 | ≤8 | ≤8 | ≤4 | 19 | 25 | 21 | ≤0.12 | 27 | ≤0.12 | ≤1 | ≤0.25 | ≤0.25 | ≤2 | ≤1 | ≤0.25 | ≤0.12 | ≤1/19 | ≤1 | ≤0.5 |
| A65 | 1 | —— | ≤0.5 | ≤8 | ≤8 | ≤4 | 15 | 24 | 21 | ≤0.12 | 26 | ≤0.12 | ≤1 | ≤0.25 | ≤0.25 | ≤2 | ≤1 | ≤0.25 | ≤0.12 | ≤1/19 | 2 | 1 |
| A67 | 1 | —— | ≤0.5 | ≤8 | ≤8 | ≤4 | 19 | 23 | —— | ≤0.12 | 31 | ≤0.12 | ≤1 | ≤0.25 | ≤0.25 | ≤2 | ≤1 | ≤0.25 | ≤0.12 | ≤1/19 | ≤1 | ≤0.5 |
| A70 | 2 | —— | ≤0.5 | ≤8 | ≤8 | ≤4 | 20 | 23 | 23 | ≤0.12 | 30 | ≤0.12 | ≤1 | ≤0.25 | ≤0.25 | ≤2 | ≤1 | ≤0.25 | ≤0.12 | ≤1/19 | 2 | 1 |
| A71 | 1 | —— | ≤0.5 | ≤8 | ≤8 | ≤4 | —— | 23 | 26 | ≤0.12 | 31 | ≤0.12 | ≤1 | ≤0.25 | ≤0.25 | ≤2 | ≤1 | ≤0.25 | ≤0.12 | ≤1/19 | 2 | ≤0.5 |
| A73 | 1 | —— | ≤0.5 | ≤8 | ≤8 | ≤4 | 16 | 23 | —— | ≤0.12 | 27 | ≤0.12 | ≤1 | ≤0.25 | ≤0.25 | ≤2 | ≤1 | ≤0.25 | ≤0.12 | ≤1/19 | ≤1 | ≤0.5 |
| A74 | 1 | —— | ≤0.5 | ≤8 | ≤8 | ≤4 | 16 | 23 | 19 | ≤0.12 | 25 | ≤0.12 | ≤1 | ≤0.25 | ≤0.25 | ≤2 | ≤1 | ≤0.25 | ≤0.12 | ≤1/19 | ≤1 | ≤0.5 |
| A75 | 1 | —— | ≤0.5 | ≤8 | ≤8 | ≤4 | 15 | 24 | —— | ≤0.12 | 28 | ≤0.12 | ≤1 | 1 | ≤0.25 | ≤2 | ≤1 | ≤0.25 | ≤0.12 | ≤1/19 | ≤1 | ≤0.5 |
| A76 | 1 | —— | ≤0.5 | ≤8 | ≤8 | ≤4 | 17 | 23 | —— | ≤0.12 | 25 | ≤0.12 | ≤1 | ≤0.25 | ≤0.25 | ≤2 | ≤1 | ≤0.25 | ≤0.12 | ≤1/19 | ≤1 | ≤0.5 |
| A77 | 1 | —— | ≤0.5 | ≤8 | ≤8 | ≤4 | 17 | 23 | 21 | ≤0.12 | 25 | ≤0.12 | ≤1 | 0.5 | ≤0.25 | ≤2 | ≤1 | ≤0.25 | ≤0.12 | ≤1/19 | 4 | ≤0.5 |
| A80 | 1 | —— | ≤0.5 | ≤8 | ≤8 | ≤4 | 15 | 23 | 20 | ≤0.12 | 24 | ≤0.12 | ≤1 | ≤0.25 | ≤0.25 | ≤2 | ≤1 | ≤0.25 | ≤0.12 | ≤1/19 | ≤1 | ≤0.5 |
| A81 | 1 | —— | ≤0.5 | ≤8 | ≤8 | ≤4 | 16 | 23 | 20 | ≤0.12 | 23 | ≤0.12 | ≤1 | ≤0.25 | ≤0.25 | ≤2 | ≤1 | ≤0.25 | ≤0.12 | ≤1/19 | ≤1 | ≤0.5 |
| A82 | 1 | —— | ≤0.5 | ≤8 | ≤8 | ≤4 | 16 | 23 | 20 | ≤0.12 | 24 | ≤0.12 | ≤1 | 0.5 | ≤0.25 | ≤2 | ≤1 | ≤0.25 | ≤0.12 | ≤1/19 | 2 | ≤0.5 |
| A95 | 1 | —— | ≤0.5 | ≤8 | ≤8 | ≤4 | 16 | 24 | 20 | ≤0.12 | 28 | ≤0.12 | ≤1 | 0.5 | ≤0.25 | ≤2 | ≤1 | ≤0.25 | ≤0.12 | ≤1/19 | 2 | ≤0.5 |
| A4 | 1 | —— | ≤0.5 | ≤8 | ≤8 | ≤4 | —— | —— | 19 | ≤0.12 | 24 | ≤0.12 | ≤1 | 1 | ≤0.25 | ≤2 | ≤1 | ≤0.25 | ≤0.12 | ≤1/19 | 2 | 1 |
| A21 | 1 | —— | ≤0.5 | ≤8 | ≤8 | ≤4 | —— | —— | 19 | ≤0.12 | 25 | ≤0.12 | ≤1 | 1 | ≤0.25 | ≤2 | ≤1 | ≤0.25 | ≤0.12 | ≤1/19 | 2 | ≤0.5 |
| A24 | 1 | —— | ≤0.5 | ≤8 | ≤8 | ≤4 | —— | —— | 21 | ≤0.12 | 25 | ≤0.12 | ≤1 | 1 | ≤0.25 | ≤2 | ≤1 | ≤0.25 | ≤0.12 | ≤1/19 | 4 | 1 |
| A47 | 1 | —— | ≤0.5 | ≤8 | ≤8 | ≤4 | —— | —— | 22 | ≤0.12 | 30 | ≤0.12 | ≤1 | 1 | ≤0.25 | ≤2 | ≤1 | ≤0.25 | ≤0.12 | ≤1/19 | 2 | ≤0.5 |
| A58 | 1 | —— | ≤0.5 | ≤8 | ≤8 | ≤4 | —— | —— | 20 | ≤0.12 | 28 | ≤0.12 | ≤1 | 1 | ≤0.25 | ≤2 | ≤1 | ≤0.25 | ≤0.12 | ≤1/19 | ≤1 | ≤0.5 |
| A66 | 1 | —— | ≤0.5 | ≤8 | ≤8 | ≤4 | —— | —— | 19 | ≤0.12 | 26 | ≤0.12 | ≤1 | 1 | ≤0.25 | ≤2 | ≤1 | ≤0.25 | ≤0.12 | ≤1/19 | ≤1 | ≤0.5 |
| A72 | 1 | —— | ≤0.5 | ≤8 | ≤8 | ≤4 | —— | —— | —— | ≤0.12 | 26 | ≤0.12 | ≤1 | 0.5 | ≤0.25 | ≤2 | ≤1 | ≤0.25 | ≤0.12 | ≤1/19 | ≤1 | 1 |
| A13 | 1 | 21 | ≤0.5 | ≤8 | ≤8 | ≤4 | 23 | 24 | 21 | ≤0.12 | 30 | ≤0.12 | ≤1 | ≤0.25 | ≤0.25 | ≤2 | ≤1 | ≤0.25 | ≤0.12 | ≤1/19 | ≤1 | ≤0.5 |
| A48 | —— | 4 | —— | —— | 28 | ≤4 | —— | —— | 23 | ≤1 | —— | ≤1 | ≤1 | ≤1 | ≤0.25 | ≤2 | ≤1 | ≤0.25 | ≤0.25 | ≤1/19 | —— | ≤0.5 |
| A60 | ≤0.5 | 19 | ≤0.5 | ≤8 | ≤8 | ≤4 | 20 | 23 | 21 | ≤0.12 | 32 | ≤0.12 | ≤1 | ≤0.25 | ≤0.25 | ≤2 | ≤1 | ≤0.25 | 0.5 | ≤1/19 | ≤1 | ≤0.5 |
| A86 | ≤0.5 | 20 | ≤0.5 | ≤8 | ≤8 | ≤4 | 20 | 23 | —— | ≤0.12 | 32 | ≤0.12 | ≤1 | ≤0.25 | ≤0.25 | ≤2 | ≤1 | ≤0.25 | 0.5 | ≤1/19 | ≤1 | ≤0.5 |
| A92 | 1 | 18 | ≤0.5 | ≤8 | ≤8 | ≤4 | 20 | 23 | 23 | ≤0.12 | 30 | ≤0.12 | ≤1 | ≤0.25 | ≤0.25 | ≤2 | ≤1 | ≤0.25 | ≤0.12 | ≤1/19 | ≤1 | ≤0.5 |
| A15 | 1 | —— | ≤0.5 | ≤8 | ≤8 | ≤4 | —— | —— | —— | 0.25 | 25 | ≤0.12 | ≤1 | ≤0.25 | ≤0.25 | ≤2 | ≤1 | ≤0.25 | ≤0.12 | ≤1/19 | 4 | ≤0.5 |
| A23 | 1 | —— | ≤0.5 | ≤8 | ≤8 | ≤4 | —— | —— | —— | 0.25 | 29 | ≤0.12 | ≤1 | ≤0.25 | ≤0.25 | ≤2 | ≤1 | ≤0.25 | 0.25 | ≤1/19 | ≤1 | ≤0.5 |
| A89 | —— | —— | ≤0.5 | ≤8 | 28 | —— | —— | —— | —— | ≤1 | —— | ≤1 | ≤1 | ≤1 | ≤0.25 | ≤2 | ≤1 | ≤0.25 | ≤0.25 | ≤1/19 | —— | ≤0.5 |
| A43 | 1 | —— | ≤0.5 | ≤8 | ≤8 | ≤4 | 18 | 23 | 8 | ≤0.12 | 30 | ≤0.12 | ≤1 | ≤0.25 | ≤0.25 | ≤2 | ≤1 | ≤0.25 | ≤0.12 | ≤1/19 | ≤1 | ≤0.5 |
| A69 | 1 | —— | ≤0.5 | ≤8 | ≤8 | ≤4 | 20 | 23 | 23 | ≤0.12 | 30 | ≤0.12 | ≤1 | ≤0.25 | ≤0.25 | ≤2 | ≤1 | ≤0.25 | ≤0.12 | ≤1/19 | ≤1 | ≤0.5 |
| A87 | 1 | —— | ≤0.5 | ≤8 | ≤8 | ≤4 | 23 | 24 | 21 | 0.25 | 27 | ≤0.12 | ≤1 | ≤0.25 | ≤0.25 | ≤2 | ≤1 | ≤0.25 | ≤0.12 | ≤1/19 | ≤1 | ≤0.5 |
| A2 | 2 | —— | ≤0.5 | ≤8 | ≤8 | ≤4 | —— | —— | —— | ≤0.12 | —— | ≤0.12 | ≤1 | ≤0.25 | ≤0.25 | ≤2 | ≤1 | ≤0.25 | ≤0.12 | ≤1/19 | 4 | 1 |
| A16 | ≤0.5 | —— | ≤0.5 | ≤8 | ≤8 | —— | —— | —— | —— | ≤0.12 | —— | —— | ≤1 | ≤0.25 | ≤0.25 | ≤2 | ≤1 | ≤0.25 | ≤0.12 | ≤1/19 | ≤1 | ≤0.5 |
| A27 | 4 | —— | —— | ≤8 | ≤8 | ≤4 | —— | —— | —— | 0.25 | 30 | ≤0.12 | ≤1 | 1 | ≤0.25 | ≤2 | 2 | ≤0.25 | ≤0.12 | —— | —— | —— |
| A33 | 1 | —— | ≤0.5 | ≤8 | ≤8 | ≤4 | —— | —— | 23 | 0.25 | 30 | ≤0.12 | ≤1 | 0.5 | ≤0.25 | ≤2 | ≤1 | ≤0.25 | ≤0.12 | ≤1/19 | ≤1 | ≤0.5 |
| A37 | 4 | —— | —— | ≤8 | ≤8 | —— | —— | —— | —— | 0.25 | 26 | ≤0.12 | ≤1 | —— | ≤0.25 | ≤2 | 2 | ≤0.25 | ≤0.12 | ≤1/19 | 4 | —— |
| A55 | 2 | —— | —— | ≤8 | ≤8 | ≤4 | —— | —— | —— | ≤0.12 | 26 | ≤0.12 | ≤1 | ≤0.25 | ≤0.25 | ≤2 | ≤1 | ≤0.25 | ≤0.12 | —— | —— | —— |
| A57 | 2 | —— | ≤0.5 | ≤8 | ≤8 | ≤4 | —— | —— | 19 | 0.25 | 25 | ≤0.12 | ≤1 | ≤0.25 | ≤0.25 | ≤2 | ≤1 | ≤0.25 | ≤0.12 | ≤1/19 | 4 | 1 |
| A93 | 1 | —— | ≤0.5 | ≤8 | ≤8 | ≤4 | —— | —— | 19 | 0.25 | 28 | ≤0.12 | ≤1 | 1 | ≤0.25 | ≤2 | ≤1 | ≤0.25 | ≤0.12 | ≤1/19 | 2 | 2 |
| B10 | 1 | —— | ≤0.5 | ≤8 | ≤8 | ≤4 | 18 | 6 | 20 | ≤0.12 | 32 | ≤0.12 | ≤1 | ≤0.25 | ≤0.25 | ≤2 | ≤1 | ≤0.25 | ≤0.12 | ≤1/19 | 2 | ≤0.5 |
| B17 | 1 | —— | ≤0.5 | ≤8 | ≤8 | ≤4 | 17 | 15 | 19 | ≤0.12 | 26 | ≤0.12 | ≤1 | ≤0.25 | ≤0.25 | ≤2 | ≤1 | ≤0.25 | ≤0.12 | ≤1/19 | 2 | ≤0.5 |
| B46 | 1 | —— | 2 | ≤8 | ≤8 | ≤4 | 8 | 28 | 21 | 0.25 | 28 | ≤0.12 | ≤1 | 1 | ≤0.25 | ≤2 | ≤1 | ≤0.25 | ≤0.12 | ≤1/19 | 2 | ≤0.5 |
| B47 | 1 | —— | ≤0.5 | ≤8 | ≤8 | ≤4 | —— | 6 | 12 | ≤0.12 | 26 | ≤0.12 | ≤1 | ≤0.25 | ≤0.25 | ≤2 | ≤1 | ≤0.25 | ≤0.12 | ≤1/19 | 2 | ≤0.5 |
| B48 | 1 | —— | ≤0.5 | ≤8 | ≤8 | ≤4 | —— | 20 | 21 | ≤0.12 | 6 | ≤0.12 | ≤1 | ≤0.25 | ≤0.25 | ≤2 | ≤1 | ≤0.25 | ≤0.12 | ≤1/19 | 2 | ≤0.5 |
| B50 | 1 | —— | ≤0.5 | ≤8 | ≤8 | ≤4 | 6 | 24 | 22 | ≤0.12 | 30 | ≤0.12 | ≤1 | ≤0.25 | ≤0.25 | ≤2 | ≤1 | ≤0.25 | ≤0.12 | ≤1/19 | 2 | ≤0.5 |
| B51 | 1 | —— | ≤0.5 | ≤8 | ≤8 | ≤4 | 15 | 18 | —— | ≤0.12 | 26 | ≤0.12 | ≤1 | 1 | ≤0.25 | ≤2 | ≤1 | ≤0.25 | ≤0.12 | ≤1/19 | 2 | ≤0.5 |
| B56 | 1 | —— | ≤0.5 | ≤8 | ≤8 | ≤4 | 18 | 23 | 6 | ≤0.12 | 28 | ≤0.12 | ≤1 | 0.5 | ≤0.25 | ≤2 | ≤1 | ≤0.25 | ≤0.12 | ≤1/19 | ≤1 | ≤0.5 |
| B60 | 1 | —— | ≤0.5 | ≤8 | ≤8 | ≤4 | 16 | 18 | —— | ≤0.12 | 23 | ≤0.12 | ≤1 | ≤0.25 | ≤0.25 | ≤2 | ≤1 | ≤0.25 | ≤0.12 | ≤1/19 | ≤1 | ≤0.5 |
| B64 | 1 | —— | ≤0.5 | ≤8 | ≤8 | ≤4 | 17 | 23 | 6 | ≤0.12 | 27 | ≤0.12 | ≤1 | ≤0.25 | ≤0.25 | ≤2 | ≤1 | ≤0.25 | ≤0.12 | ≤1/19 | 2 | 1 |
| B6 | 1 | —— | ≤0.5 | ≤8 | ≤8 | ≤4 | 16 | 17 | 19 | ≤0.12 | 24 | ≤0.12 | ≤1 | ≤0.25 | ≤0.25 | ≤2 | ≤1 | ≤0.25 | ≤0.12 | ≤1/19 | ≤1 | ≤0.5 |
| B9 | 1 | —— | ≤0.5 | ≤8 | ≤8 | ≤4 | 15 | 18 | 22 | ≤0.12 | 26 | ≤0.12 | ≤1 | ≤0.25 | ≤0.25 | ≤2 | ≤1 | ≤0.25 | ≤0.12 | ≤1/19 | 2 | ≤0.5 |
| B14 | ≤0.5 | —— | ≤0.5 | ≤8 | ≤8 | ≤4 | 15 | 6 | 11 | ≤0.12 | 32 | ≤0.12 | ≤1 | ≤0.25 | ≤0.25 | ≤2 | ≤1 | ≤0.25 | ≤0.12 | ≤1/19 | ≤1 | 0.5 |
| B37 | 2 | —— | ≤0.5 | ≤8 | ≤8 | ≤4 | —— | —— | 6 | ≤0.12 | 30 | ≤0.12 | ≤1 | 1 | ≤0.25 | ≤2 | ≤1 | ≤0.25 | ≤0.12 | ≤1/19 | 2 | ≤0.5 |
| B49 | 1 | —— | ≤0.5 | ≤8 | ≤8 | ≤4 | —— | —— | 6 | 0.25 | 26 | ≤0.12 | ≤1 | 1 | ≤0.25 | ≤2 | ≤1 | ≤0.25 | ≤0.12 | ≤1/19 | ≤1 | ≤0.5 |
| B55 | 1 | —— | ≤0.5 | ≤8 | ≤8 | ≤4 | —— | —— | 6 | 0.25 | 23 | ≤0.12 | ≤1 | 1 | ≤0.25 | ≤2 | ≤1 | ≤0.25 | ≤0.12 | ≤1/19 | 2 | ≤0.5 |
| B3 | 4 | 6 | ≤0.5 | ≤8 | ≤8 | ≤4 | 20 | 23 | 24 | ≤0.12 | 28 | ≤0.12 | ≤1 | ≤0.25 | ≤0.25 | ≤2 | 8 | ≤0.25 | ≤0.12 | ≥16/304 | ≤1 | ≤0.5 |
| B11 | 4 | 6 | ≤0.5 | 16 | ≤8 | ≤4 | 17 | 6 | 6 | 0.5 | 6 | 20 | 2 | ≤0.25 | ≤0.25 | ≤2 | ≤1 | ≤0.25 | 0.5 | ≤1/19 | ≤1 | ≤0.5 |
| B13 | 1 | 6 | ≤0.5 | 16 | ≤8 | ≤4 | 16 | 23 | 24 | ≤0.12 | 28 | ≤0.12 | ≤1 | ≤0.25 | ≤0.25 | ≤2 | ≤1 | ≤1 | ≤0.12 | ≥16/304 | ≤1 | ≤0.5 |
| B15 | ≤0.5 | 20 | ≤0.5 | ≤8 | ≤8 | ≤4 | 21 | 23 | 20 | ≤0.12 | 30 | ≤0.12 | ≤1 | ≤0.25 | ≤0.25 | ≤2 | ≤1 | 4 | 8 | ≤1/19 | 2 | ≤0.5 |
| B19 | 1 | 6 | ≤0.5 | 16 | 16 | ≤4 | 15 | 6 | 6 | 0.5 | 6 | 20 | 2 | ≤0.25 | ≤0.25 | ≤2 | ≤1 | ≤0.25 | 0.5 | ≤1/19 | ≤1 | ≤0.5 |
| B25 | 1 | 15 | ≤0.5 | ≤8 | ≤8 | ≤4 | 16 | 19 | 18 | 0.25 | 25 | ≤0.12 | ≤1 | ≤0.25 | ≤0.25 | ≤2 | ≤1 | ≤0.25 | ≤0.12 | ≤1/19 | ≤1 | ≤0.5 |
| B36 | 8 | 21 | ≤0.5 | ≤8 | ≤8 | ≤4 | 22 | 23 | 22 | ≤0.12 | 32 | ≤0.12 | ≤1 | ≤0.25 | ≤0.25 | ≤2 | ≤1 | 4 | 8 | ≤1/19 | 4 | ≤0.5 |
| B41 | ≥16 | 26 | ≤0.5 | ≤8 | ≤8 | ≤4 | 26 | 25 | 23 | ≤0.12 | 28 | ≤0.12 | ≤1 | ≤0.25 | ≤0.25 | ≤2 | ≤1 | ≤0.25 | 0.5 | ≤1/19 | 4 | ≤0.5 |
| B62 | 1 | 20 | ≤0.5 | ≤8 | ≤8 | ≤4 | 22 | 23 | 4 | ≤0.12 | 23 | ≤0.12 | ≤1 | ≤0.25 | ≤0.25 | ≤2 | ≤1 | 4 | 8 | ≤1/19 | ≤1 | ≤0.5 |
| B63 | ≤0.5 | 6 | ≤0.5 | ≤8 | ≤8 | ≤4 | 15 | 20 | ≤1 | ≤0.12 | 30 | ≤0.12 | ≤1 | ≤0.25 | ≤0.25 | ≤2 | ≤1 | ≤0.25 | 0.25 | ≥16/304 | ≤1 | ≤0.5 |
| B18 | 1 | —— | ≤0.5 | ≤8 | ≤8 | ≤4 | —— | —— | 18 | 0.25 | 28 | ≤0.12 | ≤1 | ≤0.25 | ≤0.25 | ≤2 | ≤1 | ≤0.25 | ≤0.12 | ≥16/304 | 2 | ≤0.5 |
| B43 | 2 | —— | ≤0.5 | ≤8 | ≤8 | ≤4 | —— | —— | 6 | 0.5 | 14 | ≤0.12 | ≤1 | ≤0.25 | ≤0.25 | ≤2 | ≤1 | ≤0.25 | ≤0.12 | ≤1/19 | 4 | 1 |
| B53 | 1 | —— | ≤0.5 | ≤8 | ≤8 | ≤4 | —— | —— | 6 | ≤0.12 | 25 | ≤0.12 | ≤1 | 0.5 | ≤0.25 | ≤2 | ≤1 | ≤0.25 | ≤0.12 | ≤1/19 | 2 | ≤1 |
| B16 | 4 | —— | —— | ≤8 | ≤8 | ≤4 | 6 | —— | 35 | ≤0.12 | 32 | ≤0.12 | ≤1 | 0.5 | ≤0.25 | ≤2 | ≤1 | ≤0.25 | 0.5 | ≤1/19 | —— | —— |
| B27 | —— | —— | ≤0.5 | ≤8 | ≤8 | 23 | —— | —— | —— | 0.75 | 23 | 26 | ≤1 | 1 | ≤0.25 | 4 | ≤1 | ≤0.25 | 0.5 | —— | 8 | ≤0.5 |
| B29 | ≥16 | 6 | ≤0.5 | ≤8 | ≤8 | ≤4 | 16 | 23 | 20 | ≤0.12 | 23 | ≤0.12 | ≤1 | 1 | ≤0.25 | ≤2 | ≤1 | ≤0.25 | ≤0.12 | ≤1/19 | 4 | 1 |
| B31 | ≤0.5 | —— | —— | ≤8 | ≤8 | ≤4 | —— | —— | —— | 8 | —— | 1 | ≥64 | ≤0.25 | ≤0.25 | ≤2 | ≤1 | 1 | 0.25 | ≤1/19 | ≤1 | —— |
| B32 | ≥16 | —— | —— | ≤8 | ≤8 | ≤4 | 6 | —— | —— | ≤0.12 | 30 | ≤0.12 | ≤1 | 1 | ≤0.25 | ≤2 | —— | ≤0.25 | ≤0.12 | ≤1/19 | —— | —— |
| B39 | ≥16 | 21 | —— | ≤8 | ≤8 | ≤4 | 20 | 16 | 20 | ≤0.12 | 30 | ≤0.12 | ≤1 | 1 | ≤0.25 | ≤2 | ≤1 | ≤0.25 | ≤0.12 | ≤1/19 | —— | —— |
| B40 | 2 | —— | —— | ≤8 | ≤8 | ≤4 | 15 | —— | —— | ≤0.12 | 30 | ≤0.12 | ≤1 | 1 | ≤0.25 | ≤2 | ≤1 | 0.5 | 0.5 | 4/76 | —— | —— |
| B44 | ≥16 | —— | —— | ≤8 | ≤8 | ≤4 | —— | —— | —— | ≤0.12 | —— | ≤0.12 | ≤1 | 1 | ≤0.25 | ≤2 | ≤1 | ≤0.25 | ≤0.12 | ≤1/19 | —— | —— |
| B52 | 4 | —— | ≤0.5 | ≤8 | ≤8 | 8 | 25 | 16 | —— | 0.5 | —— | ≤0.12 | ≤1 | ≤0.25 | ≤0.25 | ≤2 | ≤1 | ≤0.25 | ≤0.12 | ≤1/19 | 4 | ≤0.5 |
| B54 | ≥16 | 22 | —— | ≤8 | ≤8 | ≤4 | 22 | 18 | 25 | ≤0.12 | 30 | ≤0.12 | ≤1 | 1 | 1 | ≤2 | ≤1 | ≤0.25 | ≤0.12 | ≤1/19 | —— | —— |
| B57 | —— | —— | —— | —— | 33 | ≤4 | ≤2 | ≥64(MIC) | —— | 4 | —— | ≤1 | —— | ≤1 | —— | 4 | 2 | 0.5 | 0.5 | ≤1/19 | 2 | —— |
| B59 | ≤0.5 | 18 | 2 | ≤8 | ≤8 | ≤4 | 27 | 6 | —— | 2 | 20 | 8 | —— | ≤0.25 | ≤0.25 | 4 | ≤1 | ≤0.25 | ≤0.12 | ≤1/19 | 1 | ≤0.5 |
| B61 | —— | —— | —— | —— | —— | ≤4 | —— | 8 | —— | —— | —— | ≤1 | ≤1 | ≤1 | —— | ≤2 | —— | ≤0.25 | ≤0.25 | ≤1/19 | —— | —— |
| C2 | ≥16 | —— | ≤0.5 | 64 | ≥64 | 8 | 6 | 6 | 6 | 32 | 6 | ≥32 | ≥64 | 0.5 | ≤0.25 | ≤2 | ≤1 | ≥4 | ≥8 | ≥16/304 | ≥16 | 2 |
| C9 | 1 | —— | ≤0.5 | 64 | 16 | ≤4 | 12 | 6 | 6 | 4 | 6 | ≥32 | 16 | ≤0.25 | ≤0.25 | ≤2 | 8 | 2 | 1 | ≥16/304 | 2 | ≤0.5 |
| C10 | 1 | —— | ≤0.5 | ≥128 | ≥64 | 32 | 6 | 6 | 6 | 32 | 6 | ≥32 | ≥64 | ≤0.25 | ≤0.25 | ≤2 | ≤1 | 1 | 1 | ≥16/304 | 4 | 2 |
| C11 | ≥16 | —— | ≤0.5 | ≥128 | ≥64 | ≥128 | 6 | 6 | 6 | 32 | 6 | ≥32 | ≥64 | 0.5 | 1 | ≥64 | ≥16 | ≥4 | ≥8 | ≥16/304 | 8 | 2 |
| C12 | ≥16 | —— | ≤0.5 | ≥128 | ≤8 | 8 | 6 | 6 | 6 | 32 | 6 | 2 | 16 | ≤0.25 | ≤0.25 | ≤2 | ≥16 | ≥4 | ≥8 | ≥16/304 | ≥16 | 1 |
| C14 | ≥16 | —— | ≤0.5 | 16 | ≤8 | ≤4 | 6 | 6 | 6 | 0.5 | 6 | ≥32 | 2 | ≤0.25 | ≤0.25 | ≤2 | 8 | ≥4 | 4 | ≥16/304 | 8 | 2 |
| C24 | ≥16 | —— | ≤0.5 | 64 | 16 | ≤4 | 6 | 6 | 6 | 2 | 6 | ≥32 | 4 | ≤0.25 | ≤0.25 | ≤2 | 8 | 1 | 1 | ≥16/304 | ≥16 | 2 |
| C29 | 1 | —— | ≤0.5 | 16 | ≤8 | ≤4 | 15 | 6 | 6 | 8 | 6 | 2 | 16 | ≤0.25 | ≤0.25 | ≤2 | ≤1 | 1 | 0.5 | ≥16/304 | 2 | ≤0.5 |
| C34 | ≥16 | —— | ≤0.5 | 64 | ≤8 | 16 | 6 | 6 | 6 | 32 | 6 | ≥32 | 16 | ≤0.25 | ≤0.25 | 4 | ≥16 | ≥4 | ≥8 | ≥16/304 | 4 | 2 |
| C37 | ≥16 | —— | ≤0.5 | 32 | 16 | 8 | 6 | 6 | —— | 0.5 | —— | 2 | ≤1 | 0.5 | ≤0.25 | ≤2 | 2 | 1 | 1 | ≥16/304 | ≥16 | 2 |
| C39 | ≥16 | —— | ≤0.5 | ≥128 | ≥64 | ≥128 | 6 | 6 | 6 | 32 | 6 | ≥32 | ≥64 | ≤0.25 | ≤0.25 | ≤2 | 8 | ≥4 | ≥8 | ≥16/304 | ≥16 | 2 |
| C41 | 4 | —— | ≤0.5 | 16 | ≤8 | 8 | 15 | 6 | 6 | 32 | 6 | 4 | 16 | ≤0.25 | ≤0.25 | ≤2 | ≤1 | ≥4 | ≥8 | 4/76 | 4 | 2 |
| C47 | ≥16 | —— | ≤0.5 | 64 | 16 | 32 | 6 | 6 | 6 | 1 | 6 | 2 | 4 | ≤0.25 | ≤0.25 | ≤2 | 4 | ≥4 | ≥8 | ≥16/304 | ≥16 | 2 |
| C50 | ≥16 | —— | ≤0.5 | 16 | ≤8 | 8 | 6 | 6 | 16 | ≥64 | 6 | 2 | ≥64 | ≤0.25 | ≤0.25 | ≤2 | ≤1 | ≥4 | ≥8 | ≥16/304 | ≥16 | 2 |
| C51 | ≥16 | —— | ≤0.5 | 16 | ≤8 | 16 | 6 | 6 | 13 | 0.5 | —— | ≤0.12 | ≤1 | ≤0.25 | ≤0.25 | ≤2 | 2 | ≥4 | ≥8 | ≥16/304 | ≥16 | ≥8 |
| C58 | ≥16 | —— | ≤0.5 | 64 | 16 | ≤4 | 6 | 6 | 6 | 16 | 6 | ≥32 | 16 | ≤0.25 | ≤0.25 | ≤2 | 8 | ≥4 | ≥8 | ≥16/304 | 8 | 2 |
| C59 | 1 | —— | ≤0.5 | 64 | 16 | 8 | 6 | 6 | 6 | ≥64 | 6 | ≥32 | ≥64 | ≤0.25 | ≤0.25 | ≤2 | 8 | ≥4 | 4 | ≤1/19 | 2 | 1 |
| C61 | ≥16 | —— | ≤0.5 | 64 | 32 | 8 | 6 | 6 | —— | 4 | 6 | ≥32 | 2 | ≤0.25 | ≤0.25 | ≤2 | ≤1 | 1 | 0.5 | ≥16/304 | 8 | 2 |
| C71 | ≥16 | —— | ≤0.5 | 64 | 16 | 8 | 6 | 6 | —— | 16 | 6 | ≥32 | 16 | ≤0.25 | ≤0.25 | ≤2 | 8 | ≥4 | 4 | ≥16/304 | ≥16 | 2 |
| C73 | ≥16 | —— | ≤0.5 | ≤8 | ≤8 | ≤4 | 15 | 21 | —— | 0.5 | —— | ≤0.12 | ≤1 | ≤0.25 | ≤0.25 | ≤2 | 8 | ≥4 | 4 | ≥16/304 | ≥16 | 2 |
| C74 | 1 | —— | ≤0.5 | 16 | 16 | ≤4 | 20 | 6 | —— | 32 | 13 | ≥32 | ≥64 | ≤0.25 | ≤0.25 | ≤2 | 8 | ≥4 | 0.5 | ≤1/19 | 4 | 1 |
| C77 | ≥16 | —— | ≤0.5 | 32 | 16 | 8 | 6 | 6 | —— | 4 | —— | ≥32 | ≤1 | ≤0.25 | ≤0.25 | ≤2 | ≤1 | 1 | 1 | ≥16/304 | 8 | 2 |
| C79 | ≥16 | —— | ≤0.5 | 32 | 16 | ≤4 | 6 | 6 | —— | 16 | 6 | ≥32 | 16 | ≤0.25 | ≤0.25 | ≤2 | 8 | ≥4 | 1 | ≥16/304 | ≥16 | 2 |
| C83 | ≥16 | —— | ≤0.5 | ≥128 | 16 | 32 | 6 | 6 | —— | 32 | 6 | ≥32 | 16 | ≤0.25 | ≤0.25 | ≤2 | 8 | 1 | 0.5 | ≥16/304 | ≥16 | 2 |
| C90 | ≥16 | —— | ≤0.5 | ≥128 | ≥64 | 32 | 6 | 6 | 6 | 8 | 6 | ≥32 | ≥64 | ≤0.25 | ≤0.25 | ≤2 | 4 | 2 | 1 | ≥16/304 | ≥16 | 2 |
| NO. | DOX | AMP | COL | TCC | CSL | TZP | SAM | CZO | CAZ | CRO | FEP | ATM | IPM | MEM | AMK | TOB | CIP | LVX | SXT | MNO | TGC |  |
| C1 | ≤0.5 | 6 | ≤0.5 | ≤8 | ≤8 | ≤4 | 15 | 17 | ≤0.12 | 23 | ≤0.12 | ≤1 | ≤0.25 | ≤0.25 | ≤2 | ≤1 | ≥4 | ≥8 | ≤1/19 | ≤1 | ≤0.5 |  |
| C3 | ≥16 | 6 | ≤0.5 | ≥128 | ≥64 | ≥128 | 6 | 6 | ≥64 | 6 | 16 | ≥64 | ≤0.25 | ≤0.25 | ≤2 | ≥16 | ≥4 | ≥8 | ≥16/304 | ≥16 | ≤0.5 |  |
| C4 | 8 | 6 | ≤0.5 | 16 | 16 | ≤4 | 10 | 6 | 0.5 | 6 | 2 | 2 | ≤0.25 | ≤0.25 | ≤2 | ≤1 | ≥4 | ≥8 | ≥16/304 | ≤1 | ≤0.5 |  |
| C6 | ≥16 | 6 | ≤0.5 | 16 | ≤8 | ≤4 | 11 | 6 | ≥64 | 6 | 16 | 16 | ≤0.25 | ≤0.25 | ≤2 | 8 | ≥4 | ≥8 | ≥16/304 | 8 | ≤0.5 |  |
| C7 | 1 | 6 | ≤0.5 | 16 | 16 | ≤4 | 6 | 6 | 4 | 6 | 16 | 4 | 0.5 | ≤0.25 | 4 | ≤1 | ≥4 | ≥8 | ≥16/304 | ≤1 | ≤0.5 |  |
| C8 | 1 | 6 | ≤0.5 | ≥128 | ≥64 | 8 | 6 | 6 | 8 | 6 | ≥32 | 16 | ≤0.25 | ≤0.25 | ≤2 | ≥16 | ≥4 | ≥8 | ≤1/19 | ≤1 | ≤0.5 |  |
| C18 | ≥16 | 6 | ≤0.5 | 16 | ≤8 | ≤4 | 15 | 6 | ≥64 | 28 | ≤0.12 | ≤1 | ≤0.25 | ≤0.25 | ≤2 | 8 | ≥4 | ≥8 | ≥16/304 | ≥16 | ≤0.5 |  |
| C20 | 8 | 6 | ≤0.5 | 16 | ≤8 | ≤4 | 12 | 6 | ≤0.12 | 36 | ≤0.12 | ≤1 | ≤0.25 | ≤0.25 | ≤2 | ≤1 | ≥4 | ≥8 | ≤1/19 | 8 | ≤0.5 |  |
| C21 | 1 | 6 | ≤0.5 | 32 | 16 | ≤4 | 6 | 6 | 4 | 6 | 16 | 4 | ≤0.25 | ≤0.25 | ≤2 | ≤1 | ≥4 | ≥8 | ≤1/19 | 2 | ≤0.5 |  |
| C23 | ≥16 | 6 | ≤0.5 | 16 | ≤8 | ≤4 | 6 | 6 | 8 | 6 | 16 | 16 | ≤0.25 | ≤0.25 | ≥64 | ≥16 | ≥4 | ≥8 | ≥16/304 | ≥16 | ≤0.5 |  |
| C26 | ≥16 | 6 | ≤0.5 | ≥128 | 16 | ≥128 | 6 | 6 | ≥64 | 6 | ≥32 | ≥64 | ≤0.25 | ≤0.25 | ≤2 | 8 | ≥4 | ≥8 | ≥16/304 | ≥16 | ≤0.5 |  |
| C27 | 2 | 6 | ≤0.5 | ≥128 | ≥64 | ≥128 | 6 | 6 | 32 | 6 | ≥32 | ≥64 | ≤0.25 | ≤0.25 | ≤2 | ≥16 | ≥4 | ≥8 | ≤1/19 | 4 | ≤0.5 |  |
| C28 | ≥16 | 6 | ≤0.5 | ≥128 | 32 | ≥128 | 6 | 6 | ≥64 | 6 | 8 | 16 | ≤0.25 | ≤0.25 | ≤2 | 8 | ≥4 | ≥8 | ≥16/304 | 8 | ≤0.5 |  |
| C30 | 2 | 6 | ≤0.5 | ≥128 | 32 | 64 | 6 | 6 | ≥64 | 6 | ≥32 | ≥64 | 0.5 | ≤0.25 | ≤2 | 8 | ≥4 | ≥8 | 2/38 | 2 | ≤0.5 |  |
| C31 | ≥16 | 6 | ≤0.5 | ≤8 | 16 | ≤4 | 6 | 6 | 1 | 6 | 4 | ≤1 | ≤0.25 | ≤0.25 | ≥64 | ≥16 | ≥4 | ≥8 | ≥16/304 | ≥16 | ≤0.5 |  |
| C32 | ≥16 | 6 | ≤0.5 | ≥128 | 32 | 8 | 6 | 6 | 4 | 6 | 16 | 4 | ≤0.25 | ≤0.25 | 4 | ≤1 | ≥4 | ≥8 | ≤1/19 | ≥16 | ≤0.5 |  |
| C33 | ≥16 | 6 | ≤0.5 | 32 | 16 | ≤4 | 6 | 6 | 32 | 6 | 16 | ≥64 | ≤0.25 | ≤0.25 | ≤2 | ≥16 | ≤0.25 | 0.5 | ≤1/19 | ≥16 | ≤0.5 |  |
| C36 | ≥16 | 6 | ≤0.5 | 16 | ≤8 | ≤4 | 11 | 16 | 0.25 | 28 | ≤0.12 | ≤1 | ≤0.25 | ≤0.25 | ≤2 | 8 | ≥4 | ≥8 | ≥16/304 | ≥16 | ≤0.5 |  |
| C38 | 1 | 6 | ≤0.5 | 16 | 16 | ≤4 | 6 | 6 | 4 | —— | 4 | 4 | ≤0.25 | ≤0.25 | ≤2 | ≤1 | ≥4 | ≥8 | ≥16/304 | ≤1 | ≤0.5 |  |
| C40 | ≥16 | 6 | 8 | 64 | 16 | ≤4 | 11 | 6 | 32 | 6 | 16 | ≥64 | ≤0.25 | ≤0.25 | ≤2 | ≥16 | ≥4 | ≥8 | ≥16/304 | ≥16 | ≤0.5 |  |
| C44 | ≥16 | 6 | ≤0.5 | 16 | ≤8 | ≤4 | 10 | 15 | 4 | 29 | 1 | ≤1 | ≤0.25 | ≤0.25 | ≤2 | ≤1 | 1 | 0.5 | ≥16/304 | 4 | ≤0.5 |  |
| C45 | ≤0.5 | 6 | ≤0.5 | 32 | 16 | ≤4 | 6 | 6 | 32 | 6 | 16 | ≥64 | ≤0.25 | ≤0.25 | ≤2 | 8 | ≥4 | ≥8 | ≤1/19 | ≤1 | ≤0.5 |  |
| C46 | ≥16 | 6 | ≤0.5 | 16 | ≤8 | ≤4 | 6 | 6 | 16 | 6 | 16 | 16 | ≤0.25 | ≤0.25 | 4 | ≥16 | ≥4 | ≥8 | ≥16/304 | 4 | ≤0.5 |  |
| C52 | ≥16 | 6 | ≤0.5 | 64 | 16 | 8 | 6 | 6 | ≥64 | —— | ≥32 | ≥64 | ≤0.25 | ≤0.25 | ≤2 | ≥16 | ≥4 | ≥8 | ≥16/304 | ≥16 | 2 |  |
| C54 | ≥16 | 6 | ≤0.5 | ≥128 | 16 | 64 | 6 | 6 | 32 | —— | 16 | ≥64 | ≤0.25 | ≤0.25 | 4 | ≥16 | ≥4 | ≥8 | ≥16/304 | ≥16 | ≤0.5 |  |
| C55 | ≥16 | 6 | ≤0.5 | ≥128 | ≥64 | ≥128 | 6 | 6 | ≥64 | —— | ≥32 | ≥64 | ≤0.25 | ≤0.25 | ≥64 | ≥16 | ≥4 | ≥8 | ≥16/304 | ≥16 | ≤0.5 |  |
| C56 | ≥16 | 6 | ≤0.5 | 16 | 16 | ≤4 | 6 | 6 | 8 | 6 | ≥32 | 16 | ≤0.25 | ≤0.25 | ≤2 | ≤1 | ≥4 | ≥8 | ≤1/19 | 4 | ≤0.5 |  |
| C57 | ≥16 | 6 | ≤0.5 | 16 | 16 | ≤4 | 6 | 6 | 0.5 | —— | 4 | 2 | ≤0.25 | ≤0.25 | ≤2 | ≤1 | ≥4 | ≥8 | ≥16/304 | 8 | ≤0.5 |  |
| C60 | ≥16 | 6 | ≤0.5 | ≥128 | 16 | ≤4 | 6 | 6 | 4 | —— | 16 | 4 | ≤0.25 | ≤0.25 | ≤2 | ≤1 | ≥4 | ≥8 | ≥16/304 | 8 | ≤0.5 |  |
| C62 | ≥16 | 6 | ≤0.5 | 16 | 16 | ≤4 | 6 | 6 | 32 | —— | 16 | 32 | ≤0.25 | ≤0.25 | ≤2 | 8 | ≥4 | ≥8 | ≥16/304 | ≥16 | ≤0.5 |  |
| C63 | ≥16 | 6 | ≤0.5 | 16 | 16 | 16 | 6 | 6 | ≥64 | —— | ≥32 | ≥64 | ≤0.25 | ≤0.25 | ≤2 | 8 | 1 | 1 | ≥16/304 | ≥16 | 2 |  |
| C66 | 1 | 6 | ≤0.5 | 16 | ≤8 | ≤4 | 13 | 16 | 0.25 | 30 | ≤0.12 | ≤1 | ≤0.25 | ≤0.25 | ≤2 | ≤1 | ≥4 | ≥8 | ≤1/19 | ≤1 | ≤0.5 |  |
| C67 | ≤0.5 | 6 | ≤0.5 | 16 | ≤8 | ≤4 | 6 | 6 | 4 | 6 | 16 | 2 | ≤0.25 | ≤0.25 | ≤2 | ≥16 | ≥4 | 4 | ≥16/304 | ≤1 | ≤0.5 |  |
| C69 | ≥16 | 6 | ≤0.5 | ≥128 | 32 | ≥128 | 6 | 6 | ≥64 | 6 | ≥32 | ≥64 | ≤0.25 | ≤0.25 | ≤2 | ≤1 | ≥4 | ≥8 | ≤1/19 | 8 | ≤0.5 |  |
| C70 | 8 | 6 | ≤0.5 | ≤8 | ≤8 | ≤4 | 16 | 6 | 8 | 6 | 2 | 4 | ≤0.25 | ≤0.25 | ≤2 | ≤1 | ≤0.25 | 0.5 | ≥16/304 | ≤1 | ≤0.5 |  |
| C72 | 1 | 6 | ≤0.5 | ≤8 | ≤8 | ≤4 | 15 | 18 | ≤0.12 | 6 | ≤0.12 | ≤1 | ≤0.25 | ≤0.25 | ≤2 | ≤1 | ≥4 | ≥8 | ≤1/19 | 2 | 2 |  |
| C75 | ≥16 | 6 | ≤0.5 | ≥128 | 32 | ≥128 | 6 | 6 | ≥64 | —— | ≥32 | ≥64 | ≤0.25 | ≤0.25 | ≤2 | 8 | ≥4 | ≥8 | ≥16/304 | 4 | ≤0.5 |  |
| C76 | 4 | 6 | ≤0.5 | 16 | ≤8 | ≤4 | 16 | 6 | ≥64 | —— | 16 | 16 | ≤0.25 | ≤0.25 | ≤2 | 8 | ≥4 | ≥8 | ≥16/304 | ≤1 | ≤0.5 |  |
| C78 | 1 | 10 | ≤0.5 | ≥128 | 16 | 64 | 10 | 6 | ≥64 | 6 | ≥32 | ≥64 | ≤0.25 | ≤0.25 | ≤2 | ≤1 | ≥4 | ≥8 | ≤1/19 | ≤1 | ≤0.5 |  |
| C89 | ≥16 | 6 | ≤0.5 | 32 | 16 | ≤4 | 6 | 6 | 0.5 | 6 | 16 | 2 | ≤0.25 | ≤0.25 | ≤2 | 8 | ≤0.25 | 0.5 | ≤1/19 | 4 | ≤0.5 |  |
| C5 | ≥16 | 6 | —— | ≤8 | ≤8 | ≤4 | 6 | 6 | 0.5 | 6 | 16 | ≤1 | 21 | ≤0.25 | ≤2 | 8 | 2 | 1 | ≥16/304 | —— | —— |  |
| C13 | ≥16 | —— | ≤0.5 | ≥128 | 32 | ≥128 | —— | —— | ≥64 | 6 | ≥32 | ≥64 | 0.5 | ≤0.25 | ≤2 | ≥16 | ≥4 | ≥8 | ≥16/304 | ≥16 | 2 |  |
| C15 | ≥16 | —— | —— | 16 | 32 | ≤4 | 6 | —— | 2 | 6 | 2 | ≤1 | 0.5 | ≤0.25 | ≤2 | 8 | ≥4 | ≥8 | ≥16/304 | ≥16 | —— |  |
| C16 | 2 | 6 | ≤0.5 | 64 | ≤8 | 16 | 6 | 6 | 4 | 25 | ≤0.12 | ≤1 | 1 | ≤0.25 | 4 | 8 | ≥4 | ≥8 | ≤1/19 | 4 | ≤0.5 |  |
| C17 | ≥16 | —— | ≤0.5 | 16 | ≤8 | ≤4 | —— | —— | 0.5 | 6 | 2 | ≤1 | 1 | ≤0.25 | ≤2 | 8 | 1 | 1 | ≥16/304 | 4 | 2 |  |
| C19 | ≥16 | —— | ≤0.5 | 16 | ≤8 | ≤4 | 11 | 12 | ≤0.12 | 28 | ≤0.12 | ≤1 | ≤0.25 | ≤0.25 | ≤2 | 8 | ≥4 | ≥8 | ≥16/304 | 8 | 1 |  |
| C22 | ≥16 | —— | —— | ≤8 | ≤8 | ≤4 | 6 | —— | ≤0.12 | 30 |  | ≤1 | 1 | ≤0.25 | ≤2 | ≤1 | ≥4 | 0.5 | ≥16/304 | ≥16 | —— |  |
| C35 | 4 | 6 | —— | ≤8 | ≤8 | ≤4 | 6 | 6 | 2 | 6 | ≤0.12 | ≤1 | 1 | ≤0.25 | ≤2 | 2 | ≥4 | 4 | ≥16/304 | —— | —— |  |
| C42 | 1 | —— | —— | ≥128 | ≥64 | 32 | —— | —— | 4 | 6 | ≥32 | 16 | —— | 1 | 8 | ≥16 | ≥4 | ≥8 | ≤1/19 | ≤1 | ≤0.5 |  |
| C43 | ≥16 | —— | ≤0.5 | ≥128 | 32 | 64 | —— | —— | ≥64 | 6 | ≥32 | ≥64 | 0.5 | ≤0.25 | ≤2 | 8 | ≥4 | ≥8 | ≥16/304 | ≥16 | ≥8 |  |
| C48 | ≥16 | —— | ≤0.5 | ≥128 | 16 | 16 | —— | —— | 32 | —— | 1 | 32 | ≤0.25 | ≤0.25 | 4 | ≥16 | ≥4 | 4 | ≥16/304 | ≥16 | ≥8 |  |
| C49 | 2 | —— | ≤0.5 | ≥128 | ≥64 | ≥128 | —— | —— | ≥64 | 6 | 2 | ≥64 | ≤0.25 | ≤0.25 | ≤2 | ≤0.25 | ≤0.25 | ≤0.12 | ≤1/19 | 4 | 1 |  |
| C64 | 4 | —— | ≤0.5 | 64 | ≥64 | ≥128 | —— | —— | ≥64 | —— | 8 | ≥64 | 1 | 1 | ≤2 | ≤1 | ≤0.25 | ≤0.12 | ≤1/19 | 8 | 2 |  |
| C65 | ≥16 | 6 | —— | ≤8 | ≤8 | ≤4 | 6 | 6 | 2 | 24 | ≤0.12 | ≤1 | 1 | ≤0.25 | 4 | ≥16 | ≤0.25 | 0.5 | ≥16/304 | —— | —— |  |
| C68 | ≥16 | —— | ≤0.5 | 16 | ≤8 | ≤4 | —— | —— | 0.5 | 26 | ≤0.12 | ≤1 | ≤0.25 | ≤0.25 | ≤2 | ≤1 | ≥4 | ≥8 | ≥16/304 | ≥16 | 1 |  |
| C80 | 8 | —— | ≤0.5 | ≥128 | 32 | ≥128 | —— | —— | ≥64 | 6 | 2 | 16 | ≤0.25 | ≤0.25 | ≤2 | ≤1 | 0.5 | 1 | ≤1/19 | ≥16 | 2 |  |
| C81 | ≥16 | —— | ≤0.5 | 16 | ≤8 | 8 | —— | —— | 0.5 | —— | ≤0.12 | ≤1 | ≤0.25 | ≤0.25 | ≤2 | ≤1 | ≥4 | ≥8 | ≥16/304 | ≥16 | 2 |  |
| C82 | ≥16 | 6 | —— | ≤8 | ≤8 | ≤4 | 10 | 6 | 2 | 20 | ≤0.12 | ≤1 | 1 | ≤0.25 | ≤2 | 8 | 1 | 0.5 | ≥16/304 | —— | —— |  |
| C84 | ≥16 | —— | —— | ≤8 | ≤8 | ≤4 | 6 | —— | ≤0.12 | 30 | —— | ≤1 | 1 | ≤0.25 | ≤2 | ≤1 | 1 | 0.5 | ≤1/19 | 8 | —— |  |
| C85 | ≥16 | 6 | —— | ≤8 | ≤8 | ≤4 | 6 | 6 | ≤0.12 | 6 | 2 | ≤1 | 1 | ≤0.25 | ≤2 | ≥16 | ≥4 | ≥8 | ≥16/304 | —— | —— |  |
| C91 | ≥16 | 6 | ≤0.5 | ≥128 | ≥64 | ≥128 | 6 | 6 | 32 | 6 | ≥32 | ≥64 | 0.5 | ≤0.25 | 16 | ≥16 | ≥4 | ≥8 | 4/76 | ≥16 | 2 |  |
| D3 | ≥16 | —— | ≤0.5 | ≥128 | ≥64 | ≥128 | 6 | 6 | ≥64 | 6 | ≥32 | ≥64 | ≥16 | ≥16 | ≥64 | ≥16 | ≥4 | ≥8 | ≥16/304 | ≥16 | 2 |  |
| D7 | ≥16 | —— | ≤0.5 | ≥128 | ≥64 | ≥128 | 6 | 6 | 6 | 6 | ≥32 | ≥64 | ≥16 | ≥16 | ≥64 | ≥16 | ≥4 | ≥8 | ≥16/304 | ≥16 | 2 |  |
| D8 | ≥16 | —— | ≤0.5 | ≥128 | ≥64 | ≥128 | 6 | 6 | 6 | 6 | ≥32 | ≥64 | ≥16 | ≥16 | ≥64 | ≥16 | ≥4 | ≥8 | ≥16/304 | ≥16 | 2 |  |
| D10 | ≥16 | —— | ≤0.5 | ≥128 | ≥64 | ≥128 | 6 | 6 | 6 | 6 | ≥32 | ≥64 | ≥16 | ≥16 | ≥64 | ≥16 | ≥4 | ≥8 | ≤1/19 | 8 | 2 |  |
| D22 | ≥16 | —— | ≤0.5 | ≥128 | ≥64 | ≥128 | 6 | 6 | 6 | 6 | ≥32 | ≥64 | ≥16 | ≥16 | ≥64 | ≥16 | ≥4 | ≥8 | ≥16/304 | ≥16 | 2 |  |
| D26 | ≥16 | —— | ≤0.5 | ≥128 | ≥64 | ≥128 | 6 | 6 | ≥64 | 6 | ≥32 | ≥64 | ≥16 | ≥16 | ≤2 | ≤1 | ≥4 | ≥8 | ≤1/19 | ≥16 | 2 |  |
| D27 | ≥16 | —— | ≤0.5 | ≥128 | ≥64 | ≥128 | 6 | 6 | ≥64 | 6 | ≥32 | ≥64 | ≥16 | ≥16 | ≤2 | 8 | ≥4 | ≥8 | ≥16/304 | ≥16 | 2 |  |
| D31 | ≥16 | —— | ≤0.5 | ≥128 | ≥64 | ≥128 | 6 | 6 | ≥64 | —— | ≥32 | ≥64 | ≥16 | ≥16 | ≥64 | ≥16 | ≥4 | ≥8 | ≥16/304 | ≥16 | ≥8 |  |
| D36 | ≥16 | —— | ≤0.5 | ≥128 | 16 | ≥128 | 6 | 6 | 8 | 6 | 4 | ≥64 | ≥16 | ≥16 | ≤2 | 8 | 0.5 | 1 | ≤1/19 | ≤1 | ≤0.5 |  |
| D42 | 1 | —— | ≤0.5 | ≥128 | ≥64 | ≥128 | 6 | 6 | 32 | 6 | ≥32 | ≥64 | ≥16 | ≥16 | ≤2 | 8 | ≥4 | ≥8 | ≤1/19 | ≤1 | ≤0.5 |  |
| D44 | ≥16 | —— | ≤0.5 | ≥128 | ≥64 | ≥128 | 6 | 6 | ≥64 | 6 | ≥32 | ≤1 | ≥16 | ≥16 | 4 | ≥16 | ≥4 | ≥8 | ≥16/304 | ≥16 | ≥8 |  |
| D45 | 2 | —— | ≤0.5 | ≥128 | ≥64 | ≥128 | 6 | 6 | ≥64 | 6 | ≥32 | ≥64 | ≥16 | 8 | ≥64 | ≥16 | ≥4 | ≥8 | ≥16/304 | 2 | ≤0.5 |  |
| D46 | 2 | —— | ≤0.5 | ≥128 | ≥64 | ≥128 | —— | —— | 32 | —— | ≥32 | ≥64 | 8 | ≥16 | ≤2 | 4 | ≥4 | ≥8 | ≤1/19 | 4 | 1 |  |
| D53 | ≥16 | —— | ≤0.5 | ≥128 | ≥64 | ≥128 | 6 | 6 | ≥64 | 6 | ≥32 | ≥64 | ≥16 | ≥16 | ≤2 | ≥16 | ≥4 | ≥8 | ≥16/304 | ≥16 | 2 |  |
| D56 | 1 | —— | ≤0.5 | ≥128 | ≥64 | ≥128 | 6 | 6 | ≥64 | 6 | ≥32 | ≥64 | ≥16 | ≥16 | ≤2 | 4 | ≤0.25 | ≤0.12 | ≥16/304 | 2 | 1 |  |
| D58 | ≥16 | —— | ≤0.5 | ≥128 | ≥64 | ≥128 | 6 | 6 | ≥64 | 6 | ≥32 | ≥64 | ≥16 | ≥16 | ≥64 | ≥16 | ≥4 | ≥8 | ≥16/304 | ≥16 | —— |  |
| D62 | ≥16 | —— | ≤0.5 | ≥128 | ≥64 | ≥128 | 6 | 6 | ≥64 | 6 | ≥32 | ≥64 | ≥16 | ≥16 | ≥64 | ≥16 | ≥4 | ≥8 | ≤1/19 | 8 | 2 |  |
| D64 | ≥16 | —— | ≤0.5 | ≥128 | ≥64 | ≥128 | 6 | 6 | ≥64 | 6 | ≥32 | ≥64 | ≥16 | ≥16 | ≥64 | ≥16 | ≥4 | ≥8 | ≥16/304 | ≥16 | 2 |  |
| D66 | ≥16 | —— | ≤0.5 | ≥128 | ≥64 | ≥128 | 6 | 6 | ≥64 | 6 | ≥32 | ≥64 | ≥16 | ≥16 | ≥64 | ≥16 | ≥4 | ≥8 | ≥16/304 | 8 | 2 |  |
| D69 | ≥16 | —— | ≤0.5 | ≥128 | ≥64 | ≥128 | 6 | 6 | ≥64 | 6 | ≥32 | ≥64 | ≥16 | ≥16 | ≥64 | ≥16 | ≥4 | ≥8 | ≥16/304 | ≥16 | 2 |  |
| D71 | ≥16 | —— | ≤0.5 | ≥128 | ≥64 | ≥128 | 6 | 6 | ≥64 | 6 | ≥32 | ≥64 | ≥16 | ≥16 | 64 | ≥16 | ≥4 | ≥8 | ≥16/304 | 8 | 2 |  |
| D74 | 8 | —— | ≤0.5 | ≥128 | ≥64 | ≥128 | 6 | 6 | ≥64 | 6 | ≥32 | ≥64 | ≥16 | ≥16 | 4 | ≥16 | ≥4 | ≥8 | ≤1/19 | 2 | 2 |  |
| D80 | ≥16 | —— | ≤0.5 | ≥128 | ≥64 | ≥128 | 6 | 6 | ≥64 | 6 | ≥32 | ≥64 | ≥16 | ≥16 | ≥64 | ≥16 | ≥4 | ≥8 | ≤1/19 | 8 | 2 |  |
| D84 | 4 | —— | ≤0.5 | ≥128 | ≥64 | ≥128 | 6 | 6 | ≥64 | 6 | ≥32 | ≥64 | ≥16 | ≥16 | ≥64 | ≥16 | ≥4 | ≥8 | ≤1/19 | 8 | 2 |  |
| D85 | ≥16 | —— | ≤0.5 | ≥128 | ≥64 | ≥128 | 6 | 6 | ≥64 | 6 | ≥32 | ≥64 | ≥16 | ≥16 | ≥64 | ≥16 | ≥4 | ≥8 | ≥16/304 | ≥16 | 4 |  |
| D87 | ≥16 | —— | ≤0.5 | ≥128 | ≥64 | ≥128 | 6 | 6 | ≥64 | 6 | ≥32 | ≥64 | ≥16 | ≥16 | ≥64 | ≥16 | ≥4 | ≥8 | ≥16/304 | 8 | 2 |  |
| D90 | 2 | —— | ≤0.5 | ≥128 | ≥64 | ≥128 | 6 | —— | ≥64 | —— | ≥32 | ≥64 | ≥16 | ≥16 | ≥64 | ≥16 | ≥4 | ≥8 | ≤1/19 | 8 | 2 |  |
| D91 | ≥16 | —— | ≤0.5 | ≥128 | ≥64 | ≥128 | 6 | 6 | 32 | 6 | ≥32 | ≥64 | ≥16 | ≥16 | ≤2 | 8 | 1 | 1 | ≤1/19 | 4 | ≤0.5 |  |
| D98 | 2 | —— | ≤0.5 | ≥128 | ≥64 | ≥128 | 6 | 6 | 32 | 6 | ≥32 | ≥64 | ≥16 | ≥16 | ≥64 | ≥16 | ≥4 | ≥8 | ≥16/304 | 4 | ≤0.5 |  |
| D102 | 4 | —— | ≤0.5 | ≥128 | ≥64 | ≥128 | 6 | 6 | ≥64 | 6 | ≥32 | ≥64 | 8 | ≥16 | ≤2 | ≤1 | ≤0.25 | 0.25 | ≤1/19 | 4 | 2 |  |
| D1 | ≥16 | 6 | ≤0.5 | ≥128 | ≥64 | ≥128 | 6 | 6 | ≥32 | 6 | ≥32 | ≥64 | ≥16 | ≥16 | ≥64 | ≥16 | ≥4 | ≥8 | ≥16/304 | ≥16 | 2 |  |
| D12 | ≥16 | —— | ≤0.5 | ≥128 | ≥64 | ≥128 | 6 | 6 | ≥64 | 6 | ≥32 | ≥64 | ≥16 | ≥16 | ≤2 | ≥16 | ≥4 | ≥8 | ≥16/304 | 4 | ≤0.5 |  |
| D38 | ≥16 | —— | ≤0.5 | ≥128 | ≥64 | ≥128 | —— | —— | ≥64 | —— | ≥32 | ≥64 | ≥16 | ≥16 | ≤2 | 8 | ≥4 | ≥8 | ≥16/304 | ≥16 | ≤0.5 |  |
| D43 | ≥16 | 6 | ≤0.5 | ≥128 | ≥64 | ≥128 | 6 | 6 | ≥32 | 6 | ≥32 | ≥64 | ≥16 | ≥16 | ≥64 | ≥16 | ≥4 | ≥8 | ≥16/304 | ≥16 | 2 |  |
| D49 | ≥16 | —— | ≤0.5 | ≥128 | ≥64 | ≥128 | —— | —— | ≥64 | —— | ≥32 | ≥64 | ≥16 | ≥16 | ≤2 | ≤1 | ≥4 | ≥8 | ≥16/304 | ≥16 | 2 |  |
| D50 | ≥16 | 6 | ≤0.5 | ≥128 | ≥64 | ≥128 | 6 | 6 | ≥64 | 6 | ≥32 | 32 | ≥16 | 8 | ≤2 | ≥16 | ≥4 | ≥8 | ≥16/304 | 8 | ≤0.5 |  |
| D88 | ≥16 | —— | ≤0.5 | ≥128 | ≥64 | ≥128 | 6 | 6 | ≥64 | 6 | ≥32 | ≥64 | ≥16 | ≥16 | ≥64 | ≥16 | ≥4 | ≥8 | ≥16/304 | 4 | ≤0.5 |  |
| D13 | ≥16 | —— | ≤0.5 | ≥128 | ≥64 | ≥128 | —— | —— | ≥64 | 6 | ≥32 | 16 | ≥16 | ≥16 | ≤2 | 8 | ≥4 | 4 | ≥16/304 | ≥16 | ≥8 |  |
| D14 | ≥16 | —— | ≤0.5 | ≥128 | ≥64 | ≥128 | —— | —— | ≥64 | 6 | ≥32 | 16 | ≥16 | ≥16 | ≤2 | 4 | ≥4 | 4 | ≥16/304 | ≥16 | ≥8 |  |
| D23 | ≥16 | —— | ≤0.5 | ≥128 | ≥64 | ≥128 | —— | —— | ≥64 | 6 | ≥32 | ≤1 | ≥16 | ≥16 | ≤2 | ≤1 | ≥4 | ≥8 | ≥16/304 | ≥16 | ≥8 |  |
| D33 | ≥16 | —— | ≤0.5 | ≥128 | ≥64 | ≥128 | —— | —— | ≥64 | 6 | ≥32 | ≥64 | ≥16 | ≥16 | ≤2 | 4 | ≥4 | 4 | ≥16/304 | 4 | 1 |  |
| D34 | ≥16 | —— | ≤0.5 | ≥128 | ≥64 | ≥128 | —— | —— | ≥64 | 6 | ≥32 | 16 | ≥16 | ≥16 | ≤2 | 8 | ≥4 | 4 | ≥16/304 | ≥16 | ≥8 |  |
| D35 | ≥16 | —— | ≤0.5 | ≥128 | ≥64 | ≥128 | —— | —— | ≥64 | 6 | 16 | ≥64 | ≥16 | ≥16 | ≤2 | 8 | 1 | 1 | ≥16/304 | ≥16 | 1 |  |
| D76 | 8 | —— | ≤0.5 | ≥128 | ≥64 | ≥128 | —— | —— | ≥64 | 6 | ≥32 | ≥64 | 8 | ≥16 | ≤2 | ≥16 | ≥4 | ≥8 | ≤1/19 | 4 | 2 |  |
| D81 | —— | —— | —— | —— | —— | ≥128 | —— | —— | ≥64 | —— | ≥64 | ≤1 | ≥16 | ≥16 | 4 | ≤1 | ≥4 | ≥8 | ≤1/19 | —— | —— |  |
| D92 | 8 | —— | ≤0.5 | ≥128 | 32 | ≥128 | —— | —— | ≥64 | 6 | 16 | ≤1 | ≥16 | ≥16 | ≤2 | ≤1 | ≤0.25 | 0.5 | ≥16/304 | 4 | 1 |  |
| D2 | ≥16 | 6 | ≤0.5 | ≥128 | ≥64 | ≥128 | 6 | 6 | ≥64 | 6 | ≥32 | 16 | ≥16 | ≥16 | 16 | ≥16 | ≥4 | ≥8 | ≥16/304 | 4 | ≤0.5 |  |
| D9 | ≥16 | —— | ≤0.5 | ≥128 | 32 | ≤4 | —— | —— | 32 | —— | 4 | ≥64 | 4 | 4 | 4 | ≥16 | 1 | 1 | ≥16/304 | ≥16 | 2 |  |
| D20 | ≥16 | —— | ≤0.5 | ≥128 | ≥64 | ≥128 | —— | —— | 32 | —— | 4 | ≥64 | ≥16 | 4 | 4 | ≥16 | 1 | 1 | ≥16/304 | ≥16 | 2 |  |
| D29 | ≥16 | —— | —— | ≥128 | ≥64 | ≥128 | 6 | —— | ≥64 | 6 | ≥32 | 16 | ≥16 | ≥16 | 16 | ≥16 | ≥4 | ≥8 | ≥16/304 | —— | —— |  |
| D30 | ≥16 | —— | ≤0.5 | ≥128 | ≥64 | ≥128 | —— | —— | ≥64 | 6 | ≥32 | ≥64 | ≥16 | ≥16 | ≤2 | 8 | ≥4 | ≥8 | ≥16/304 | ≥16 | 2 |  |
| D39 | 4 | —— | ≤0.5 | ≥128 | ≥64 | ≥128 | 6 | 6 | 32 | 6 | ≥32 | ≥64 | 8 | ≥16 | ≤2 | 4 | ≥4 | 4 | ≤1/19 | 8 | 2 |  |
| D40 | ≥16 | —— | —— | ≥128 | ≥64 | 16 | 6 | —— | ≥64 | 6 | 1 | ≤1 | ≥16 | ≥16 | ≤2 | 8 | ≥4 | 4 | ≥16/304 | ≥16 | —— |  |
| D47 | 1 | —— | —— | ≥128 | 32 | ≤4 | —— | —— | 32 | —— | 4 | ≥64 | 4 | 4 | 4 | ≥16 | 1 | 1 | ≥16/304 | —— | —— |  |
| D51 | 1 | —— | —— | ≥128 | 32 | ≤4 | —— | —— | 32 | —— | 4 | ≥64 | 4 | 4 | 4 | ≥16 | 1 | 1 | ≥16/304 | —— | —— |  |
| D52 | 4 | —— | —— | ≥128 | 32 |  | —— | —— | ≥64 | 26 | ≥32 | ≥64 | —— | ≥16 | 8 | ≥16 | 0.25 | 0.25 | 4/76 | 2 | ≥8 |  |
| D73 | 8 | 6 | —— | ≥128 | ≥64 | ≥128 | 6 | 6 | ≥64 | 6 | ≥32 | ≥64 | ≥16 | ≥16 | ≥64 | ≥16 | ≥8 | ≥8 | ≤1/19 | 4 | 2 |  |
| D94 | 8 | —— | —— | ≥128 | ≥64 | ≥128 | ≥64 | 6 | ≥64 | —— | ≥32 | ≥64 | ≥16 | ≥16 | ≥64 | ≥16 | ≥8 | ≥8 | ≥16/304 | ≥16 | 2 |  |

DOX: Doxycycline, COL: Colistin, TZP: Piperacillin/Tazobactam, TCC: Ticarcillin/clavulanate, CSL: cefoperazone/sulbactam, SAM: Ampicillin/Sulbactam, CAZ: Ceftazidime, FEP: Cefepime, MEM: Meropenem, IPM: Imipenem, TOB: Tobramycin, Amikacin, LVX: Levofloxacin, CIP: Ciprofloxacin, SXT: Paediatric Compound Sulfamethoxazole, MNO: Minocycline, TGC: Tigecycline, CRO: Ceftriaxone, PIP: Piperacillin, CZO, Cefazolin, ATM: Aztreonam, GEN: Gentamicin, AMP: Ampicillin, CXM: Cefuroxim, CAZ: Ceftazidime
