# Supplementary material for: Difference analysis and characteristics of incompatibility group plasmid replicons in gram-negative bacteria with different antimicrobial phenotypes in Henan, China
Source: BMC Microbiol. 2024 Feb 19;24:64. doi: 10.1186/s12866-024-03212-9 (PMC10875880; doi:10.1186/s12866-024-03212-9)
Supplement: Supplementary file 2 — Supplementary Material 2: Antimicrobial susceptibility testing of E. coli in Group C [file 12866_2024_3212_MOESM2_ESM.docx]

Table 2 Antimicrobial susceptibility testing of *E. coli* in Group C

| Number | DOX | AMP | COL | TCC | CSL | TZP | SAM | CZO | CAZ | CRO | FEP | ATM | IPM | MEM | AMK | TOB | CIP | LVX | SXT | MNO | TGC |
| --- | --- | --- | --- | --- | --- | --- | --- | --- | --- | --- | --- | --- | --- | --- | --- | --- | --- | --- | --- | --- | --- |
| C1 | ≤0.5 | 6 | ≤0.5 | ≤8 | ≤8 | ≤4 | 15 | 17 | ≤0.12 | 23 | ≤0.12 | ≤1 | ≤0.25 | ≤0.25 | ≤2 | ≤1 | ≥4 | ≥8 | ≤1/19 | ≤1 | ≤0.5 |
| C3 | ≥16 | 6 | ≤0.5 | ≥128 | ≥64 | ≥128 | 6 | 6 | ≥64 | 6 | 16 | ≥64 | ≤0.25 | ≤0.25 | ≤2 | ≥16 | ≥4 | ≥8 | ≥16/304 | ≥16 | ≤0.5 |
| C4 | 8 | 6 | ≤0.5 | 16 | 16 | ≤4 | 10 | 6 | 0.5 | 6 | 2 | 2 | ≤0.25 | ≤0.25 | ≤2 | ≤1 | ≥4 | ≥8 | ≥16/304 | ≤1 | ≤0.5 |
| C6 | ≥16 | 6 | ≤0.5 | 16 | ≤8 | ≤4 | 11 | 6 | ≥64 | 6 | 16 | 16 | ≤0.25 | ≤0.25 | ≤2 | 8 | ≥4 | ≥8 | ≥16/304 | 8 | ≤0.5 |
| C7 | 1 | 6 | ≤0.5 | 16 | 16 | ≤4 | 6 | 6 | 4 | 6 | 16 | 4 | 0.5 | ≤0.25 | 4 | ≤1 | ≥4 | ≥8 | ≥16/304 | ≤1 | ≤0.5 |
| C8 | 1 | 6 | ≤0.5 | ≥128 | ≥64 | 8 | 6 | 6 | 8 | 6 | ≥32 | 16 | ≤0.25 | ≤0.25 | ≤2 | ≥16 | ≥4 | ≥8 | ≤1/19 | ≤1 | ≤0.5 |
| C18 | ≥16 | 6 | ≤0.5 | 16 | ≤8 | ≤4 | 15 | 6 | ≥64 | 28 | ≤0.12 | ≤1 | ≤0.25 | ≤0.25 | ≤2 | 8 | ≥4 | ≥8 | ≥16/304 | ≥16 | ≤0.5 |
| C20 | 8 | 6 | ≤0.5 | 16 | ≤8 | ≤4 | 12 | 6 | ≤0.12 | 36 | ≤0.12 | ≤1 | ≤0.25 | ≤0.25 | ≤2 | ≤1 | ≥4 | ≥8 | ≤1/19 | 8 | ≤0.5 |
| C21 | 1 | 6 | ≤0.5 | 32 | 16 | ≤4 | 6 | 6 | 4 | 6 | 16 | 4 | ≤0.25 | ≤0.25 | ≤2 | ≤1 | ≥4 | ≥8 | ≤1/19 | 2 | ≤0.5 |
| C23 | ≥16 | 6 | ≤0.5 | 16 | ≤8 | ≤4 | 6 | 6 | 8 | 6 | 16 | 16 | ≤0.25 | ≤0.25 | ≥64 | ≥16 | ≥4 | ≥8 | ≥16/304 | ≥16 | ≤0.5 |
| C26 | ≥16 | 6 | ≤0.5 | ≥128 | 16 | ≥128 | 6 | 6 | ≥64 | 6 | ≥32 | ≥64 | ≤0.25 | ≤0.25 | ≤2 | 8 | ≥4 | ≥8 | ≥16/304 | ≥16 | ≤0.5 |
| C27 | 2 | 6 | ≤0.5 | ≥128 | ≥64 | ≥128 | 6 | 6 | 32 | 6 | ≥32 | ≥64 | ≤0.25 | ≤0.25 | ≤2 | ≥16 | ≥4 | ≥8 | ≤1/19 | 4 | ≤0.5 |
| C28 | ≥16 | 6 | ≤0.5 | ≥128 | 32 | ≥128 | 6 | 6 | ≥64 | 6 | 8 | 16 | ≤0.25 | ≤0.25 | ≤2 | 8 | ≥4 | ≥8 | ≥16/304 | 8 | ≤0.5 |
| C30 | 2 | 6 | ≤0.5 | ≥128 | 32 | 64 | 6 | 6 | ≥64 | 6 | ≥32 | ≥64 | 0.5 | ≤0.25 | ≤2 | 8 | ≥4 | ≥8 | 2/38 | 2 | ≤0.5 |
| C31 | ≥16 | 6 | ≤0.5 | ≤8 | 16 | ≤4 | 6 | 6 | 1 | 6 | 4 | ≤1 | ≤0.25 | ≤0.25 | ≥64 | ≥16 | ≥4 | ≥8 | ≥16/304 | ≥16 | ≤0.5 |
| C32 | ≥16 | 6 | ≤0.5 | ≥128 | 32 | 8 | 6 | 6 | 4 | 6 | 16 | 4 | ≤0.25 | ≤0.25 | 4 | ≤1 | ≥4 | ≥8 | ≤1/19 | ≥16 | ≤0.5 |
| C33 | ≥16 | 6 | ≤0.5 | 32 | 16 | ≤4 | 6 | 6 | 32 | 6 | 16 | ≥64 | ≤0.25 | ≤0.25 | ≤2 | ≥16 | ≤0.25 | 0.5 | ≤1/19 | ≥16 | ≤0.5 |
| C36 | ≥16 | 6 | ≤0.5 | 16 | ≤8 | ≤4 | 11 | 16 | 0.25 | 28 | ≤0.12 | ≤1 | ≤0.25 | ≤0.25 | ≤2 | 8 | ≥4 | ≥8 | ≥16/304 | ≥16 | ≤0.5 |
| C38 | 1 | 6 | ≤0.5 | 16 | 16 | ≤4 | 6 | 6 | 4 | UN | 4 | 4 | ≤0.25 | ≤0.25 | ≤2 | ≤1 | ≥4 | ≥8 | ≥16/304 | ≤1 | ≤0.5 |
| C40 | ≥16 | 6 | 8 | 64 | 16 | ≤4 | 11 | 6 | 32 | 6 | 16 | ≥64 | ≤0.25 | ≤0.25 | ≤2 | ≥16 | ≥4 | ≥8 | ≥16/304 | ≥16 | ≤0.5 |
| C44 | ≥16 | 6 | ≤0.5 | 16 | ≤8 | ≤4 | 10 | 15 | 4 | 29 | 1 | ≤1 | ≤0.25 | ≤0.25 | ≤2 | ≤1 | 1 | 0.5 | ≥16/304 | 4 | ≤0.5 |
| C45 | ≤0.5 | 6 | ≤0.5 | 32 | 16 | ≤4 | 6 | 6 | 32 | 6 | 16 | ≥64 | ≤0.25 | ≤0.25 | ≤2 | 8 | ≥4 | ≥8 | ≤1/19 | ≤1 | ≤0.5 |
| C46 | ≥16 | 6 | ≤0.5 | 16 | ≤8 | ≤4 | 6 | 6 | 16 | 6 | 16 | 16 | ≤0.25 | ≤0.25 | 4 | ≥16 | ≥4 | ≥8 | ≥16/304 | 4 | ≤0.5 |
| C52 | ≥16 | 6 | ≤0.5 | 64 | 16 | 8 | 6 | 6 | ≥64 | UN | ≥32 | ≥64 | ≤0.25 | ≤0.25 | ≤2 | ≥16 | ≥4 | ≥8 | ≥16/304 | ≥16 | 2 |
| C54 | ≥16 | 6 | ≤0.5 | ≥128 | 16 | 64 | 6 | 6 | 32 | UN | 16 | ≥64 | ≤0.25 | ≤0.25 | 4 | ≥16 | ≥4 | ≥8 | ≥16/304 | ≥16 | ≤0.5 |
| C55 | ≥16 | 6 | ≤0.5 | ≥128 | ≥64 | ≥128 | 6 | 6 | ≥64 | UN | ≥32 | ≥64 | ≤0.25 | ≤0.25 | ≥64 | ≥16 | ≥4 | ≥8 | ≥16/304 | ≥16 | ≤0.5 |
| C56 | ≥16 | 6 | ≤0.5 | 16 | 16 | ≤4 | 6 | 6 | 8 | 6 | ≥32 | 16 | ≤0.25 | ≤0.25 | ≤2 | ≤1 | ≥4 | ≥8 | ≤1/19 | 4 | ≤0.5 |
| C57 | ≥16 | 6 | ≤0.5 | 16 | 16 | ≤4 | 6 | 6 | 0.5 | UN | 4 | 2 | ≤0.25 | ≤0.25 | ≤2 | ≤1 | ≥4 | ≥8 | ≥16/304 | 8 | ≤0.5 |
| C60 | ≥16 | 6 | ≤0.5 | ≥128 | 16 | ≤4 | 6 | 6 | 4 | UN | 16 | 4 | ≤0.25 | ≤0.25 | ≤2 | ≤1 | ≥4 | ≥8 | ≥16/304 | 8 | ≤0.5 |
| C62 | ≥16 | 6 | ≤0.5 | 16 | 16 | ≤4 | 6 | 6 | 32 | UN | 16 | 32 | ≤0.25 | ≤0.25 | ≤2 | 8 | ≥4 | ≥8 | ≥16/304 | ≥16 | ≤0.5 |
| C63 | ≥16 | 6 | ≤0.5 | 16 | 16 | 16 | 6 | 6 | ≥64 | UN | ≥32 | ≥64 | ≤0.25 | ≤0.25 | ≤2 | 8 | 1 | 1 | ≥16/304 | ≥16 | 2 |
| C66 | 1 | 6 | ≤0.5 | 16 | ≤8 | ≤4 | 13 | 16 | 0.25 | 30 | ≤0.12 | ≤1 | ≤0.25 | ≤0.25 | ≤2 | ≤1 | ≥4 | ≥8 | ≤1/19 | ≤1 | ≤0.5 |
| C67 | ≤0.5 | 6 | ≤0.5 | 16 | ≤8 | ≤4 | 6 | 6 | 4 | 6 | 16 | 2 | ≤0.25 | ≤0.25 | ≤2 | ≥16 | ≥4 | 4 | ≥16/304 | ≤1 | ≤0.5 |
| C69 | ≥16 | 6 | ≤0.5 | ≥128 | 32 | ≥128 | 6 | 6 | ≥64 | 6 | ≥32 | ≥64 | ≤0.25 | ≤0.25 | ≤2 | ≤1 | ≥4 | ≥8 | ≤1/19 | 8 | ≤0.5 |
| C70 | 8 | 6 | ≤0.5 | ≤8 | ≤8 | ≤4 | 16 | 6 | 8 | 6 | 2 | 4 | ≤0.25 | ≤0.25 | ≤2 | ≤1 | ≤0.25 | 0.5 | ≥16/304 | ≤1 | ≤0.5 |
| C72 | 1 | 6 | ≤0.5 | ≤8 | ≤8 | ≤4 | 15 | 18 | ≤0.12 | 6 | ≤0.12 | ≤1 | ≤0.25 | ≤0.25 | ≤2 | ≤1 | ≥4 | ≥8 | ≤1/19 | 2 | 2 |
| C75 | ≥16 | 6 | ≤0.5 | ≥128 | 32 | ≥128 | 6 | 6 | ≥64 | UN | ≥32 | ≥64 | ≤0.25 | ≤0.25 | ≤2 | 8 | ≥4 | ≥8 | ≥16/304 | 4 | ≤0.5 |
| C76 | 4 | 6 | ≤0.5 | 16 | ≤8 | ≤4 | 16 | 6 | ≥64 | UN | 16 | 16 | ≤0.25 | ≤0.25 | ≤2 | 8 | ≥4 | ≥8 | ≥16/304 | ≤1 | ≤0.5 |
| C78 | 1 | 10 | ≤0.5 | ≥128 | 16 | 64 | 10 | 6 | ≥64 | 6 | ≥32 | ≥64 | ≤0.25 | ≤0.25 | ≤2 | ≤1 | ≥4 | ≥8 | ≤1/19 | ≤1 | ≤0.5 |
| C89 | ≥16 | 6 | ≤0.5 | 32 | 16 | ≤4 | 6 | 6 | 0.5 | 6 | 16 | 2 | ≤0.25 | ≤0.25 | ≤2 | 8 | ≤0.25 | 0.5 | ≤1/19 | 4 | ≤0.5 |

Minimum inhibitory concentrations (MICs) of DOX, COL, TCC, TZP, CAZ, FEP, ATM, IPM, MEM, AMK, TOB, CIP, LVX, SXT, MNO, TGC were determined and the Kirby-Bauer method of AMP, SAM, CZO, CRO, were determined. Results were interpreted according to the guideline of CLSI. Considering the absence of CLSI breakpoints for interpretation of tigecycline and colistin results, the current European Committee on Antimicrobial Susceptibility Testing (EUCAST)guidelines were used to interpret colistin (susceptible, ≤2 mg/L; resistant, ≥4 mg/L) and the Food and Drug Administration was used as the breakpoints for tigecycline (susceptible, ≤2 mg/L; resistant, ≥8 mg/L); Red indicates resistance; UN refers to clinical data not mentioned.
